# A Comprehensive Cancer-Associated MicroRNA Expression Profiling and Proteomic Analysis of Human Umbilical Cord Mesenchymal Stem Cell-Derived Exosomes

**DOI:** 10.1007/s13770-022-00450-8

**Published:** 2022-05-05

**Authors:** Ganesan Jothimani, Surajit Pathak, Suman Dutta, Asim K. Duttaroy, Antara Banerjee

**Affiliations:** 1 Department of Medical Biotechnology, Faculty of Allied Health Sciences, Chettinad Hospital and Research Institute (CHRI), Chettinad Academy of Research and Education (CARE), Kelambakkam, Chennai, Tamil Nadu 603 103 India; 2 International Institute of Innovation and Technology, DH Block, Action Area 1D, Newtown, Kolkata, West Bengal 700156 India; 3 Department of Nutrition, Institute of Basic Medical Sciences, Faculty of Medicine, University of Oslo, Oslo, Norway

**Keywords:** Mesenchymal stem cells, Exosomes, miRNA, Proteome, Gene ontology, Cancer, Stem cell therapeutics

## Abstract

**Background:**

The mesenchymal stem cells (MSCs) have enormous therapeutic potential owing to their multi-lineage differentiation and self-renewal properties. MSCs express growth factors, cytokines, chemokines, and non-coding regulatory RNAs with immunosuppressive, anti-tumor, and migratory properties. MSCs also release several anti-cancer molecules via extracellular vesicles, that act as pro-apoptotic/tumor suppressor factors. This study aimed to identify the stem cell-derived secretome that could exhibit anti-cancer properties through molecular profiling of cargos in MSC-derived exosomes.

**Methods:**

Human umbilical cord mesenchymal stem cells (hUCMSCs) were isolated from umbilical cord tissues and culture expanded. Subsequently, exosomes were isolated from hUCMSC conditioned medium and characterized by DLS, electron microscopy. Western blot for exosome surface marker protein CD63 expression was performed. The miRNA profiling of hUCMSCs and hUCMSC-derived exosomes was performed, followed by functional enrichment analysis.

**Results:**

The tri-lineage differentiation potential, fibroblastic morphology, and strong expression of pluripotency genes indicated that isolated fibroblasts are MSCs. The isolated extracellular vesicles were 133.8 ± 42.49 nm in diameter, monodispersed, and strongly expressed the exosome surface marker protein CD63. The miRNA expression profile and gene ontology (GO) depicted the differential expression patterns of high and less-expressed miRNAs that are crucial to be involved in the regulation of apoptosis. The LCMS/MS data and GO analysis indicate that hUCMSC secretomes are involved in several oncogenic and inflammatory signaling cascades.

**Conclusion:**

Primary human MSCs released miRNAs and growth factors via exosomes that are increasingly implicated in intercellular communications, and hUCMSC-exosomal miRNAs have a critical influence in regulating cell death and apoptosis of cancer cells.

**Graphical abstract:**

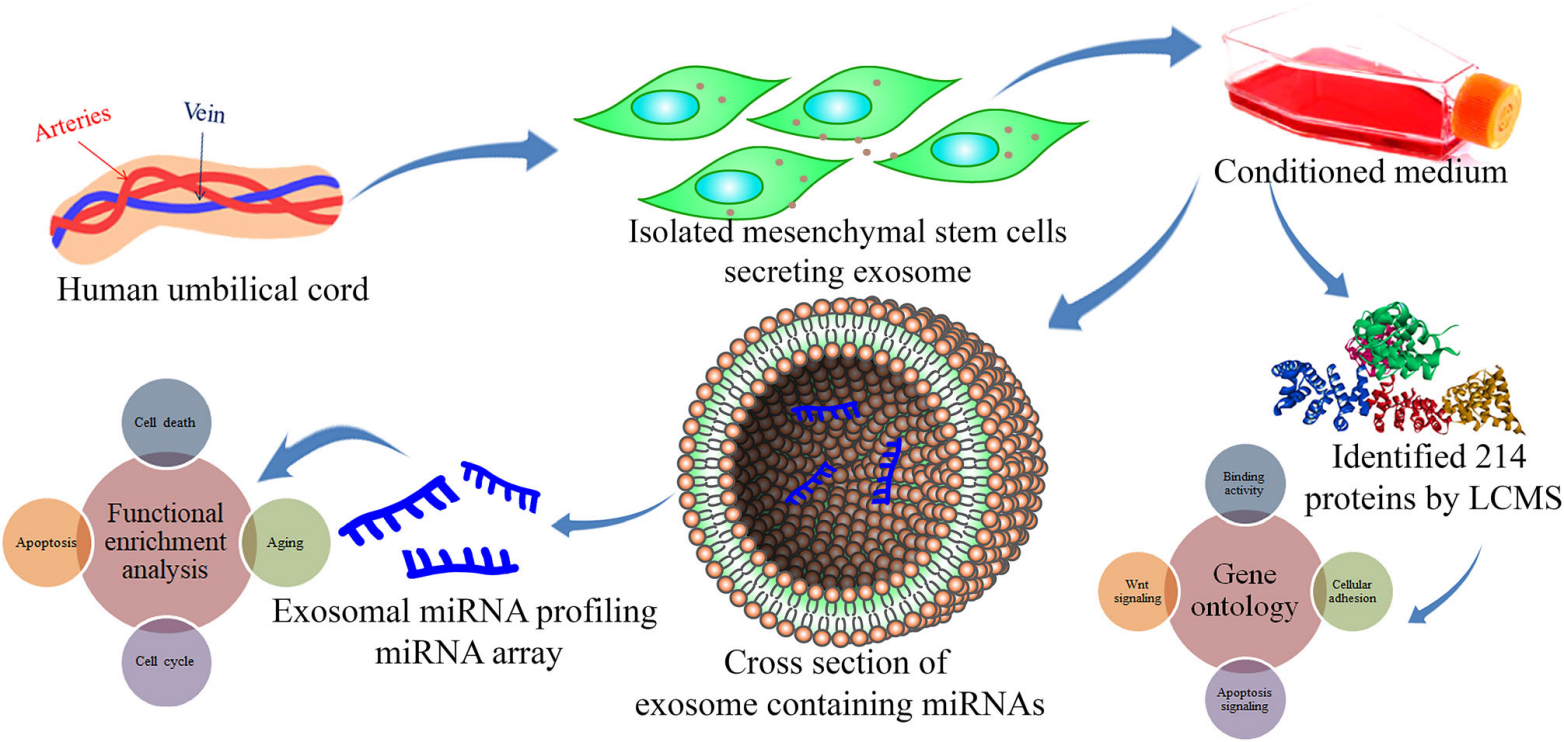

**Supplementary Information:**

The online version contains supplementary material available at 10.1007/s13770-022-00450-8.

## Introduction

The mesenchymal stem cells (MSCs) are multipotent stromal cells that can differentiate into various cellular lineages such as adipogenic, osteogenic, chondrogenic, and myogenic lineages. The MSCs actively regulate various cellular processes, including maintaining tissue homeostasis and regeneration of connective tissues. Owing to multi-lineage differentiation potential and self-renewal properties, MSCs have been demonstrated to hold enormous therapeutic potential in the field of biomedical research [1, 2]. Besides its self-renewal and differentiation capabilities, MSCs express growth factors, cytokines, chemokines, and regulatory non-coding RNAs with immunosuppressive, anti-tumor, and migratory properties that modulate the innate host cellular immune response [3, 4]. The MSCs also possess unique characteristics like the ability to migrate and home in the primary inflamed site, secrete bioactive factors, and exhibit immunosuppressive properties, fostering tumor-targeting options and future therapeutic possibilities [5]. MSC releases several non-coding RNAs and anti-cancer molecules such as tumor necrosis factor (TNF), TNF-related apoptosis-inducing ligand (TRAIL), and interferon-β (IFN-β) via exosomes that acts as pro-apoptotic or tumor suppressor factors [6–8].

Exosomes are extracellular vesicles (EVs), produced and released by all eukaryotic cell types, and are 30–200 nm in diameter. These biological nano-vesicles contain a large number of small molecules that can be transferred from one cell to another, including lipids, proteins, cytokines, microRNAs (miRNAs), mRNAs, transfer RNA (tRNA), long non-coding RNAs (lncRNAs), and mitochondrial DNAs (mtDNAs). Exosomes aid in the autocrine, paracrine and endocrine signaling by delivering different cargos to different neighboring and/or distant cells [9]. With the support of specific receptor-ligand interaction, exosomes easily communicate with their target cells to exert biological processes [10–13]. MSC-derived exosomes have gained tremendous interest since the discovery of their ability to transfer cargos to recipient cells and thereby modulate recipient cells’ geometry. Some recent studies have revealed the miRNA landscape of MSC-derived exosomes [14]. Over 938 specific proteins and 150 miRNAs have been observed and studied in detail that aid the transfer of bioactive molecules to perform both physiological and pathological processes such as organism development, epigenetic regulation, immunoregulation (involving *miR-155* and *miR-146*), and tumorigenesis (involving *miR-23b, miR-451, miR-223, miR-24, miR-125b, miR-31, miR-214,* and *miR-122*) [13, 15–17]. Considering the regulatory functions of miRNAs such as proliferation, apoptosis, development, angiogenesis, and metabolism, they can act as onco-miRs and tumor suppressor miRs depending on the target mRNA, which is briefly discussed in one of our earlier published papers [18].

According to previous reports, MSC-derived exosomes are rich in cytokines and growth factors such as TGF-1, interleukin-6 (IL-6), IL-10, and hepatocyte growth factor (HGF), all of which cumulatively contribute to immunoregulation [19]. It is also reported that the MSC-derived exosomes interact with the Hedgehog, AKT, and Wnt signaling cascades to incite cancer stem cell-like properties in the recipient cells [20–22]. However, the delivery of bone marrow mesenchymal stem cell (BM-MSC) derived exosomes have been shown to restrict cellular proliferation and induce apoptosis in various cancer models, including ovarian and liver cancer cells [23].

Similarly, it has been shown that MSC-derived exosomes inhibit vascular endothelial growth factor (VEGF) expression and control the tumor progression and angiogenesis in breast cancer cells [24]. Hence the stromal cell-derived exosomes are thought to be a double-edged sword in the TME in regulating tumorigenesis and apoptosis. Literature suggests that MSC-derived exosomes are dynamic biological elements of MSCs that carry crucial functional cargos, the reason they have gained enormous interest in cancer research [25]. These exosomes may also aid in the progression of epithelial-mesenchymal transition (EMT), promoting tumorigenicity and, in some reports elaborating towards controlling apoptosis and favoring anti-tumorigenicity. However, understanding the multifaceted interplay between MSC-derived exosomal mediators such as different proteins and miRNAs could be novel tools for diagnosis, follow-up, management, and monitoring of many diseased states. In the present study, we isolated primary MSCs from the human umbilical cord tissues and characterized the progenitor cell population with reported markers for MSC characterization. After that, we isolated extracellular vesicles from the hUCMSC-derived conditioned medium and profiled the hUCMSC-derived exosomal miRNA, performed proteome analysis of human umbilical cord-derived MSC condition medium (CM), and conducted a network analysis to identify the predominant biological processes and pathways regulated by exosomal miRNAs.

## Methods

### Isolation of human umbilical cord mesenchymal stem cell (hUCMSCs)

Umbilical cord (UC) collection and isolation were approved by the Institutional Human Ethics Committee, Chettinad Academy of Research and Education (IHEC–CARE, ethical clearance certificate number: 143/IHEC/06-18 dated 17/07/2018). Informed consent was obtained from all donors enrolled in the study.

The UC was collected in a sterile tube containing cord collection medium (PBS supplemented with 0.5% FBS), immediately after full-time delivery from mothers at the Chettinad Hospital and Research Institute (CHRI), Kelambakkam, Chennai, India. The umbilical cord was processed, and the MSCs were isolated within 6–12 h and expanded according to the protocol reported previously by Banerjee et al. [26]. In brief, the UC arteries and veins were manually dissected, followed by the UC tissues. Wharton's jelly was minced into small 2 mm diameter fragments and plated in low glucose DMEM supplemented with 20% FBS, L-glutamax, and 1% antibiotic–antimycotic solution (Gibco, Grand Island, NY, USA). The explants were incubated at 37 °C in normoxic conditions. Within 7–12 days, the UCMSCs began to migrate from the explants.

### *In vitro* characterization of hUCMSCs

#### Gene expression analysis

Total RNA was isolated from hUCMSCs using Qiazol reagent (Qiagen, Valencia, CA, USA). RNA concentration and purity were determined using nanodrop, and the cDNA was synthesized using a Eurogentec cDNA synthesis kit. The qPCR was performed with cDNA using SYBR green qPCR master mix (Takyon, Eurogentec, Saint-Jean, Belgium) for the list of genes mentioned in Table 1. Each sample was taken in triplicates for the assay. Ct values were obtained for all the genes and normalized with GAPDH to generate ΔCt values. The fold changes were calculated, and graphs were plotted using GraphPad Prism V9.

**Table 1:** Primer sequence for the list of genes analyzed in this study

Gene name	Sequence (5′–3′)
*GAPDH*	
Forward	ACAGTTGCCATGTAGACC
Reverse	TTGAGCACAGGGTACTTTA
*SOX9*	
Forward	TTCCGCGACGTGGACAT
Reverse	TCAAACTCGTTGACATCGAAGGT
*POU5F1*	
Forward	CGACCATCTGCCGCTTTGAG
Reverse	CCCCCTGTCCCCCATTCCTA
*NANOG*	
Forward	AGTCCCAAAGGCAAACAACCCACTTC
Reverse	ATCTGCTGGAGGCTGAGGTATTTCTGTCTC
*NT5E*	
Forward	TGGTCCAGGCCTATGCTTTTG
Reverse	GGGATGCTGCTGTTGAGAAGAA
*ENG*	
Forward	GCACAACCTCTGGCTGTCTTT
Reverse	ATCCTGGCTTCCCTCTTCAC
*THY1*	
Forward	AGACCCCAGTCCAGATCCAGG
Reverse	GGAGACCTGCAAGACTGTTAGC
*PECAM1*	
Forward	CACAGCAATTCCTCAGGCTA
Reverse	TTCAGCCTTCAGCATGGTAG
*PTPRC*	
Forward	CGTAATGGAAGTGCTGCAATGT
Reverse	CTGGGAGGCCTACACTTGACA
*KIT*	
Forward	GCACAATGGCACGGTTGAAT
Reverse	GGTGTGGGGATGGATTTGCT

#### Tri-lineage differentiation assay

In-vitro differentiation assay was performed on culture-expanded hUC fibroblasts between passages 1–3. The cells were passaged in MSC proliferating medium for 1–2 days followed by incubating with complete Adipogenic, Osteogenic, and Chondrocyte differentiation medium as per manufacturers protocol (HiMedia Laboratories Pvt. Ltd., India) to induce adipogenesis, osteogenesis, and chondrogenesis. The stimulation for cellular differentiation was carried out for 2–4 weeks, and the media was replaced with fresh media after every three days. After 2–4 weeks of incubation, the spent medium was removed, and ice-cold methanol was added to the cells and kept at − 20 °C for 10 min. The cells were then stained with 0.3% Oil Red O, Von Kossa, and 0.1% Toluidine blue stain, pH 2.0–2.5 (Sigma-Aldrich, St. Louis, MO, USA) as per manufacturers' recommendation. The experiment was performed in triplicates, the cells were observed under an inverted microscope (Olympus, Tokyo, Japan), and images for trilineage differentiation were captured using Leica Optica Image Viewer software.

#### Cell cycle analysis of MSCs

In addition to the stemness property, we tried to assess the proliferation and self-renewal rate of isolated MSCs through cell cycle progression analysis before going for further experiments. We performed BrdU incorporation at various time intervals to correlate self-renewability with the proliferation rate of isolated hUCMSCs. Immunocytochemistry (ICC) analysis was performed using the 5-Bromo-2-deoxyuridine (BrdU) incorporation assay to evaluate the isolated UCMSCs proliferation rate via monitoring the cell cycle progression for different time intervals (2, 4, and 6 h). BrdU is a pyrimidine analog of thymidine that is selectively incorporated into cell DNA at the S phase of the cell cycle. UC-MSCs were starved in a serum-free medium overnight to obtain synchronized cells. Approximately 2000 cells/well in culture slides (4 chambers, of BD Falcon) were then pulse-labeled by adding 10 μM BrdU for different time intervals (ranging from 1 to 6 h) and to identify the proliferating capacity of MSC at different time intervals. Mouse anti-BrdU IgG (Sigma-Aldrich) as primary antibody was added to the cells, and the plates were incubated overnight at 4 °C. After washing the cells 3 times with PBS, goat anti-mouse IgG conjugated with horseradish peroxidase (HRP) was added and incubated for 1 h at room temperature. The experiment was performed in triplicate and the images were captured using a fluorescent confocal microscope.

### Collection and preparation of conditioned medium

The isolated hUCMSCs were cultured in serum depleted medium for 3–4 days. The conditioned medium was then collected, centrifuged at 3000 rpm for 45 min to remove cell debris, and concentrated using a protein concentrator with a 10 kDa filter, and the total protein was estimated using the Bradford reagent.

### Cytokine profiling

The levels of cytokines were determined using a Custom Multi-Analyte ELISArray kit (Qiagen, # CMEH6092A) following the manufacturer's protocol. The absorptions were measured at 450, and 570 nm, and the data were normalized. The data were finally corrected by subtracting each cytokine's normalized absorbance from the negative control. The experiment was performed in duplicate and the graph was plotted with mean ± SD using GraphPad Prism V9.

### Proteome analysis of hUCMSC CM

25 µg of the total CM protein sample were separated using SDS PAGE. The samples were then subjected to in-gel trypsin digestion followed by mass spectroscopy. Briefly, the gel was washed 3 times with deionized water and stained with Coomasie brilliant blue. A side portion of the gel was used as a control band area. Using a sterile surgical blade, the gel slices were trimmed, de-stained in 50% acetonitrile (in 25 mM NH_4_HCO_3_), and incubated for 30 min with occasional vortexing until the gel became colorless and shrunk. The gel slices were dehydrated by incubating in 100% Acetonitrile for 5 min. 100 µl of Trypsin Gold (mass spectrometry grade; Cat. No. V5280; Promega, Madison, WI, USA) was added to the dried gel slices and incubated at 4 °C for 30 min. An additional 100 µl of Trypsin Gold was added, and the samples were incubated for another 90 min at 4 °C to saturate the samples with trypsin. Then 20 µl of 40 mM NH_4_HCO_3_/10% acetonitrile was added and incubated at 37 °C overnight. The supernatant was collected in a clean sterile tube and incubated for 60 min with 100 µl of 50% Acetonitrile/5% Trifluoroacetic acid, and the extracts were collected to new collection tubes. The eluted digested peptides were pooled, vacuum concentrated, loaded on the LCMS columns, and mass spectrometric analysis was carried out. The mass spectrometric data were smoothed, centroided, and converted into a mascot generic file (.mgf). The data were then analyzed with Bruker Compass Data Analysis 4.4 software, and the proteins were identified from the MASCOT database feeding the LCMS/MS output.

#### Functional enrichment analysis

The identified proteins were fed to the STRING database (www.string-db.org) for the analysis of network pathways and the Panther database (www.pantherdb.org) for the gene ontology analysis. The network pathways were trimmed with the interaction score parameter set to "highest confidence" with a value of 0.9 to provide the highest interacting proteins only, which acted as a protein hub in the network and was clustered using MCL clustering. The functional enrichment analysis was done for the gene ontology (GO) molecular function, GO biological process, GO protein class, and GO pathway analysis. The graphs were plotted for the functional enrichment analysis using GraphPad Prism V9.

### Exosome isolation

The P1 passage of hUCMSCs was cultured in an MSC proliferation medium for 2–3 days. After reaching nearly 70% confluency, the spent medium was removed, and the cells were washed with sterile PBS 5 times gently to remove any FBS-derived exosomes in the medium. Freshly prepared DMEM supplemented with exosome depleted FBS (Invitrogen, Carlsbad, CA, USA) was then added, and the cells were cultured for another 72 h. The cells were then examined for overall morphology, and the conditioned medium (CM) was collected, and exosomes were isolated using Total Exosome Isolation reagent (Invitrogen) using manufacturers protocol with minor modification. In brief, CM was centrifuged at 2000 g for 60 min to remove cell debris and other contaminating particles, followed by adding 1/2th of the total exosome isolation reagent and incubated overnight at 4˚C. After incubation, the mixture was centrifuged at 10,000 g for 1 h, and the exosome pellet was resuspended in sterile-filtered PBS and stored at −80 °C until use.

#### Characterization of hUCMSC exosomes

##### High-resolution scanning electron microscopy (HR-SEM)

10 µl of exosome preparation was loaded onto a clean cover glass and was allowed to dry at room temperature. The exosome samples were then analyzed by FEI-Quanta FEG 200F electron microscope, and photographs were taken at 20,000× and 60,000× magnifications respectively at the Sophisticated Analytical Instrument Facility (SAIF), Indian Institute of Technology (IIT-Madras).

##### Hi-resolution transmission electron microscopy (HR-TEM)

3 µl of paraformaldehyde (2%) fixed exosome sample was loaded onto gold-coated carbon formvar grids and was allowed to dry at room temperature. The grids were then washed thrice with filtered water and were then postfixed in 1% glutaraldehyde for 5 min and contrasted successively in freshly prepared 2% uranyl acetate, pH7. Observations were made with a JEOL Japan, JEM-2100 plus Hi-Resolution Transmission Electron Microscope. Images were taken with a cooled slow-scan CCD camera at a magnification of 40,000× and 100,000× respectively at the SRM Central Instrumentation Facility (SCIF), SRM Institute of Science and Technology (SRMIST).

##### Dynamic light scattering (DLS)

20 µl of the exosome sample was diluted with PBS (0.22 µm filtered and chilled). The samples were analyzed at 25 °C using a Nano ZS90, Malvern Instrument, to determine the size and structural integrity. The DLS measurements were conducted in 11 replicates, each for 50 s. The data were collected at each time point. The average intensity, volume, polydispersity index, and charge of exosomes were measured, and the data were analyzed using Zetasizer software V7.12.

##### Western blot analysis

The western blot analysis for the detection of exosomal marker protein CD63, a membrane tetraspanin was performed on hUCMSCs, hUCMSC-derived extracellular vesicles, and hUCMSC-CM. The total protein was isolated from hUCMSC and hUCMSC-derived extracellular vesicles using RIPA lysis buffer (cat. no. 20188, Millipore, Burlington, MA, USA). The concentration of protein from hUCMSC lysates, hUCMSC-derived extracellular vesicle lysates and hUCMSC-CM was quantified using Bradford reagent (cat. no. B6916, Merck, Kenilworth, NJ, USA). 30 µg of protein was electrophorized using 15% non-reducing polyacrylamide gel. The separated protein was transferred to the Immobilon PVDF membrane (cat. no. ISEQ00005, Millipore), blocked with 5% BSA for 1 h, and overnight incubation was done with Anti-CD63 antibody (cat. no. ab59479, Abcam, Cambridge, UK) at 4 °C. Then after discarding the primary antibody and the membrane was washed 3 times with TBST and incubated with HRP conjugated goat anti-mouse secondary antibody (cat. no. G21040, Invitrogen) for 1 h. The color was developed by the subsequent addition of 3,3′,5,5′-Tetramethylbenzidine (TMB) (cat. no. 1002892932, Sigma-Aldrich). The experiment was performed in triplicates and the images were captured for anlysis.

#### miRNA profiling of hUCMSCs and hUCMSC-derived exosomes

Total RNA was isolated from hUCMSCs (p1 passage) and hUCMSC-derived exosomes using miRNeasy Micro Kit (cat. no. 217084, Qiagen). The RNA concentration and purity were determined using nanodrop. 200 ng of RNA were used to synthesize miR cDNA using the miScript RT II kit (cat. no. 218160, Qiagen) as per manufacturers’ protocol. The qPCR-based miRNA array was performed on synthesized cDNA using precoated miRNA array plates -MIHS 102ZD-Human Cancer Pathway Finder (cat. no. 331221, Qiagen) and miScript SYBR green PCR kit (cat. no. 218073, Qiagen). The quantification cycle (Cq) values of target miRNAs normalized with Cq values of reference miRNAs (*SNORD61, SNOR68, SNORD72, SNORD95, SNORD96A,* and *RNU6-6P*) to calculate 2^−ΔCt^ using the data analysis program provided by Qiagen, and the graphs were plotted using Graphpad Prism V9.

#### miRNA-mRNA interaction and functional enrichment analysis

Top 35 highly expressed hUCMSC exosomal miRNAs were selected for the enrichment analysis. The miRNA names were fed as a query in the miRNet V2.0 database. The selection query parameters include *Homo sapiens*, ID type as miR Base ID, and targets as Genes from miRTarBase V8.0. The network was then curated by the Network tool "Degree filter." The cut-off value for the degree filter was set as 7.0 to identify the crucial hubs in the network. Finally, the miRNA functional enrichment analysis was performed, such as the miRNA molecular function and its associated gene ontology (GO), KEGG pathway analysis, GO Reactome analysis, and GO biological processes. The graphs are plotted using Graphpad Prism V9.

## Results

### Isolation of hUCMSC

**Fig. 1:**
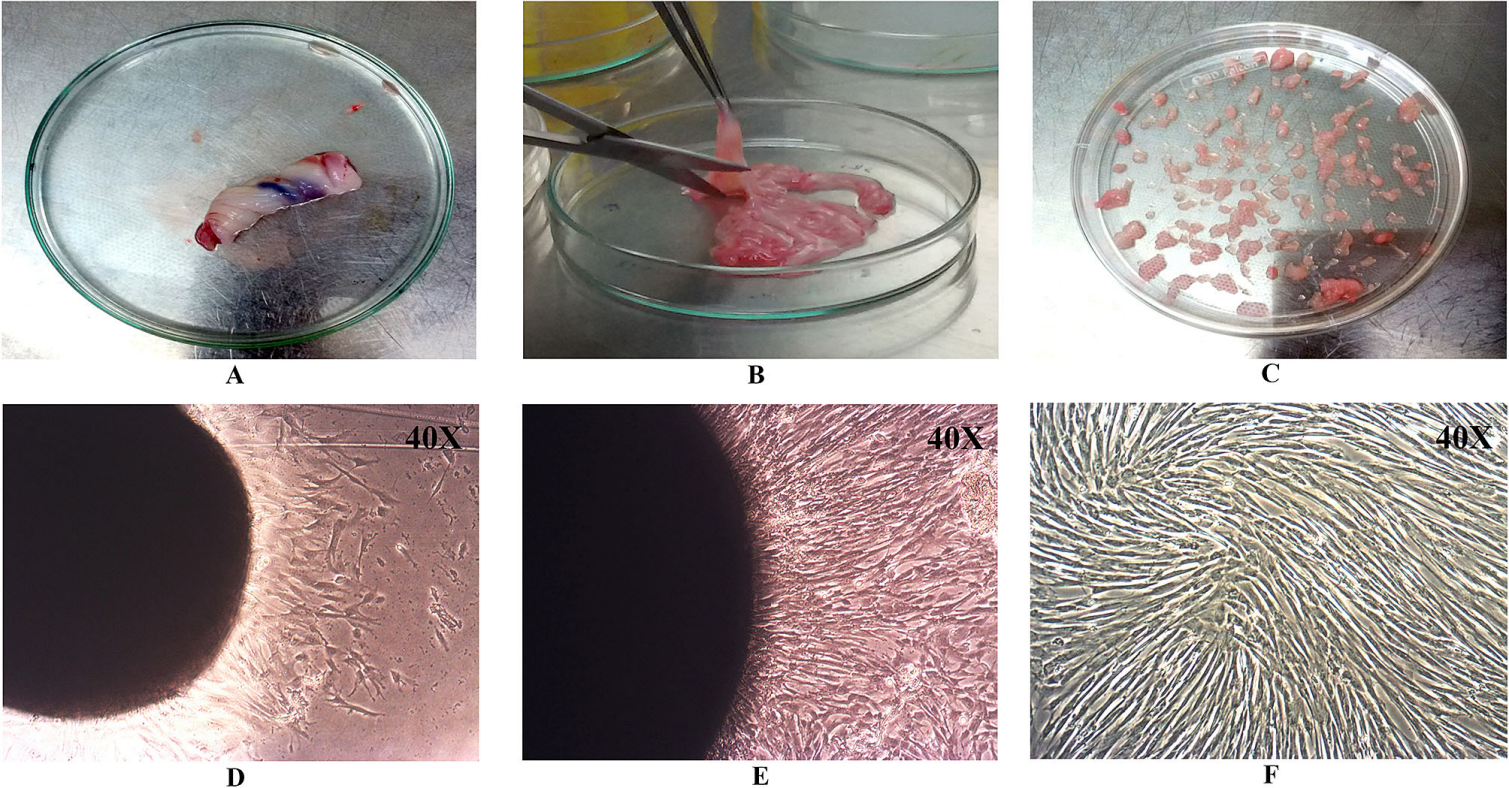
Human umbilical cord dissection and isolation of hUCMSCs. **A** human umbilical cord, **B** dissected human umbilical cord tissue, **C** Plated human umbilical cord explants, **D** mesenchymal stem cells migrated from plated explants after 6 days (#P0), **E** Confluent cultures of hUCMSCs at #P0 after 12 days, **F** Confluent cultures of hUCMSC at passage 1 (#P1)

The homogenous population of elongated, fibroblastic, and spindle-shaped cells was observed to migrate from explants after 6–7 days of culture. After 12–14 days of culture, fibroblastic colonies were observed around the tissue explants, and the density of the cells decreased with increasing distance from the colonial center. The cells resembled the MSC in morphology, as documented in our earlier studies, after successive passages. Images of UC dissection, plating the explant, hUCMSC cell release, and morphology at #P0–#P1 are shown in Fig. 1A–F.

### *In vitro* characterization of hUCMSCs

#### hUCMSC gene expression profiling

**Fig. 2:**
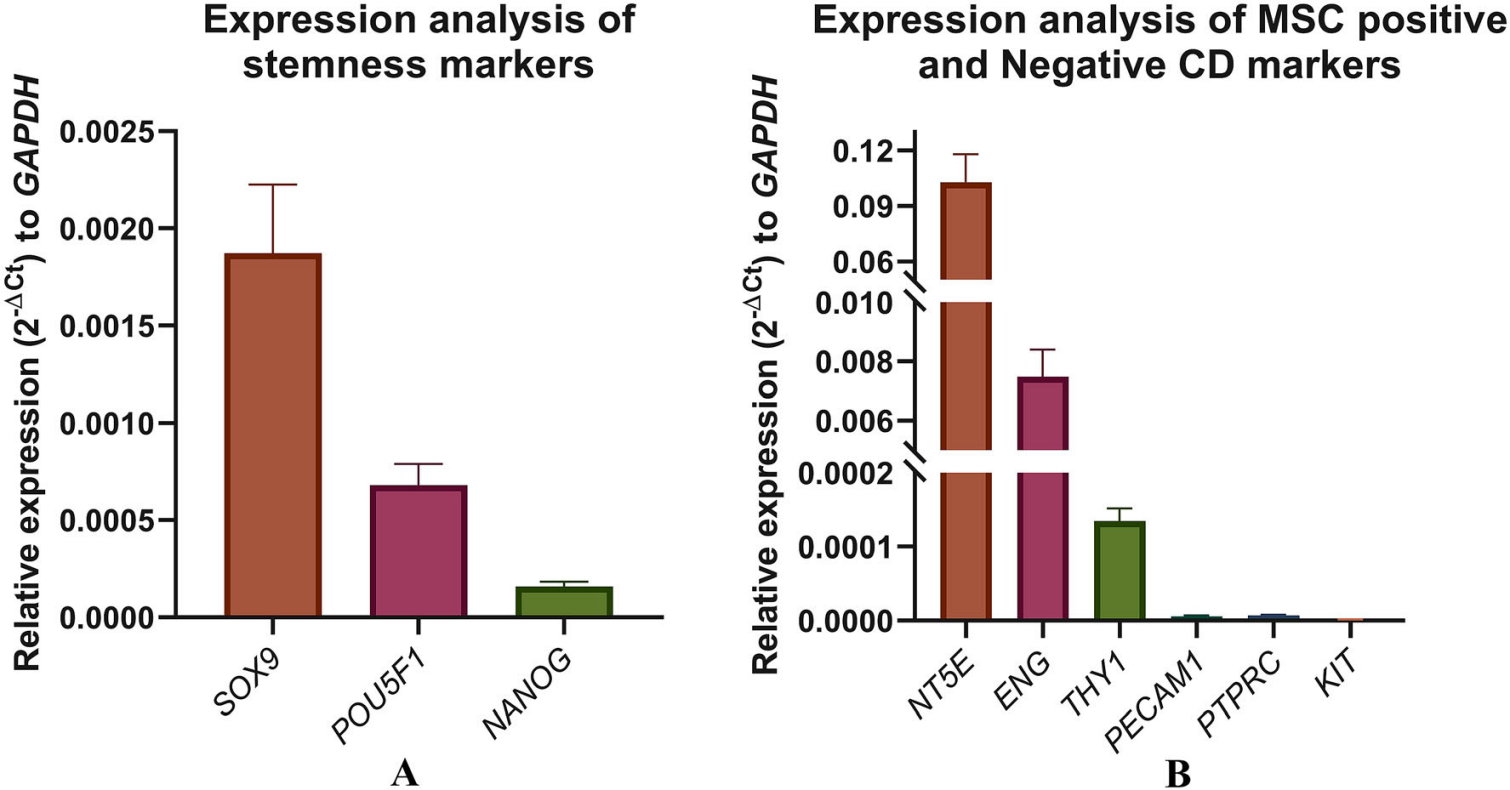
Gene expression analysis by qRT-PCR on isolated hUCMSCs. **A** Stem cell marker of pluripotency, **B** cell surface markers of lineage uncommitted or committed cells. All experiments were performed in triplicates

The qPCR expression profiling of isolated hUC fibroblasts showed a very high expression level of pluripotency markers, including *SOX9*, *POU5F1 (OCT4),* and *NANOG* and MSC positive clusters differentiation markers such as *NT5E (CD73)*, *ENG (CD105),* and *THY1 (CD90)*. On the other hand, the negative markers for MSC, including *PECAM1 (CD31)*, *PTPRC (CD45),* and *KIT (CD117),* were not expressed by the cells, as shown in Fig. 2.

#### Tri-lineage differentiation assay

3.2.2.1 Adipogenic differentiation

**Fig. 3:**
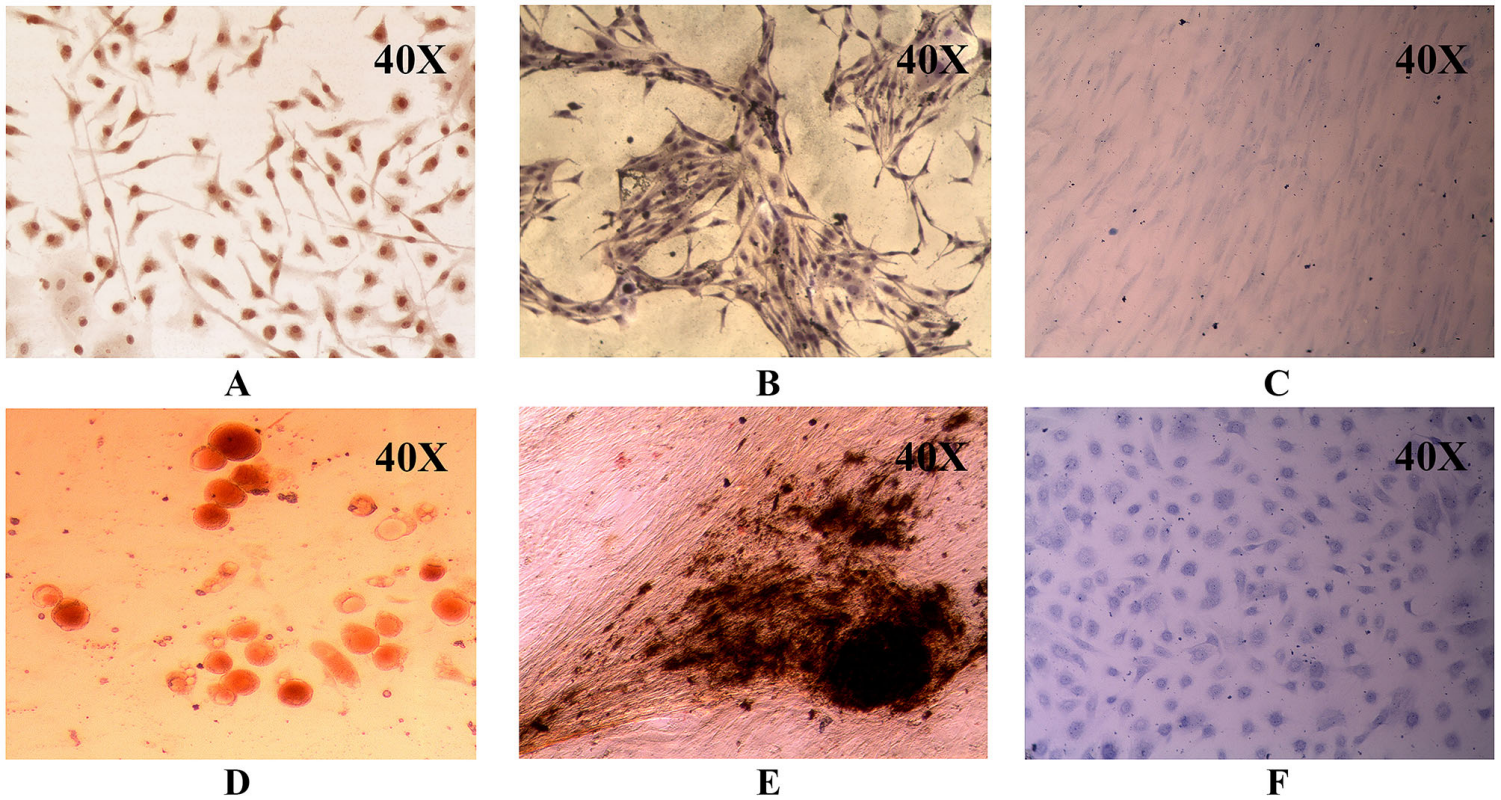
A-F hUCMSC differentiation using suitable differentiation-inducing media. Oil Red staining of **A** hUCMSCs and **D** differentiated adipocytes, Von Kossa staining of **B** hUCMSCs and **E** differentiated osteocytes, Toluidine blue staining of **C** hUCMSCs and **F** Differentiated chondrocytes

The isolated fibroblasts were cultured for two weeks supplemented with an adipogenic differentiation medium. The adipogenic characteristics were evaluated by staining the cells with Oil Red O, which stains the lipid vesicles produced in the adipocyte that appears red, and the cells were transformed into a spherical shape, which was evident when compared to undifferentiated cells cultured in standard medium, as shown in Fig. 3.

3.2.2.2 Osteogenic differentiation

After 3–4 weeks of culture in the Osteogenic differentiation medium, the differentiation pattern of the isolated cells into osteocytes was observed after staining the cells with Von Kossa stain. The calcium deposits produced by differentiated osteocytes were stained black. The cells also appeared to be morphologically elongated, confirming that the cells differentiated successfully into osteocytes, as shown in Fig. 3.

3.2.2.3 Chondrocyte differentiation

After four weeks of culturing the cells in the chondrogenic medium, the chondrocyte characteristics were evaluated by staining the cells with the toluidine blue, which targets the chondrocyte-produced proteoglycan called aggrecan, and when observed under the microscope, it appeared blue shown in Fig. 3.

#### Cell cycle analysis of MSC

**Fig. 4:**
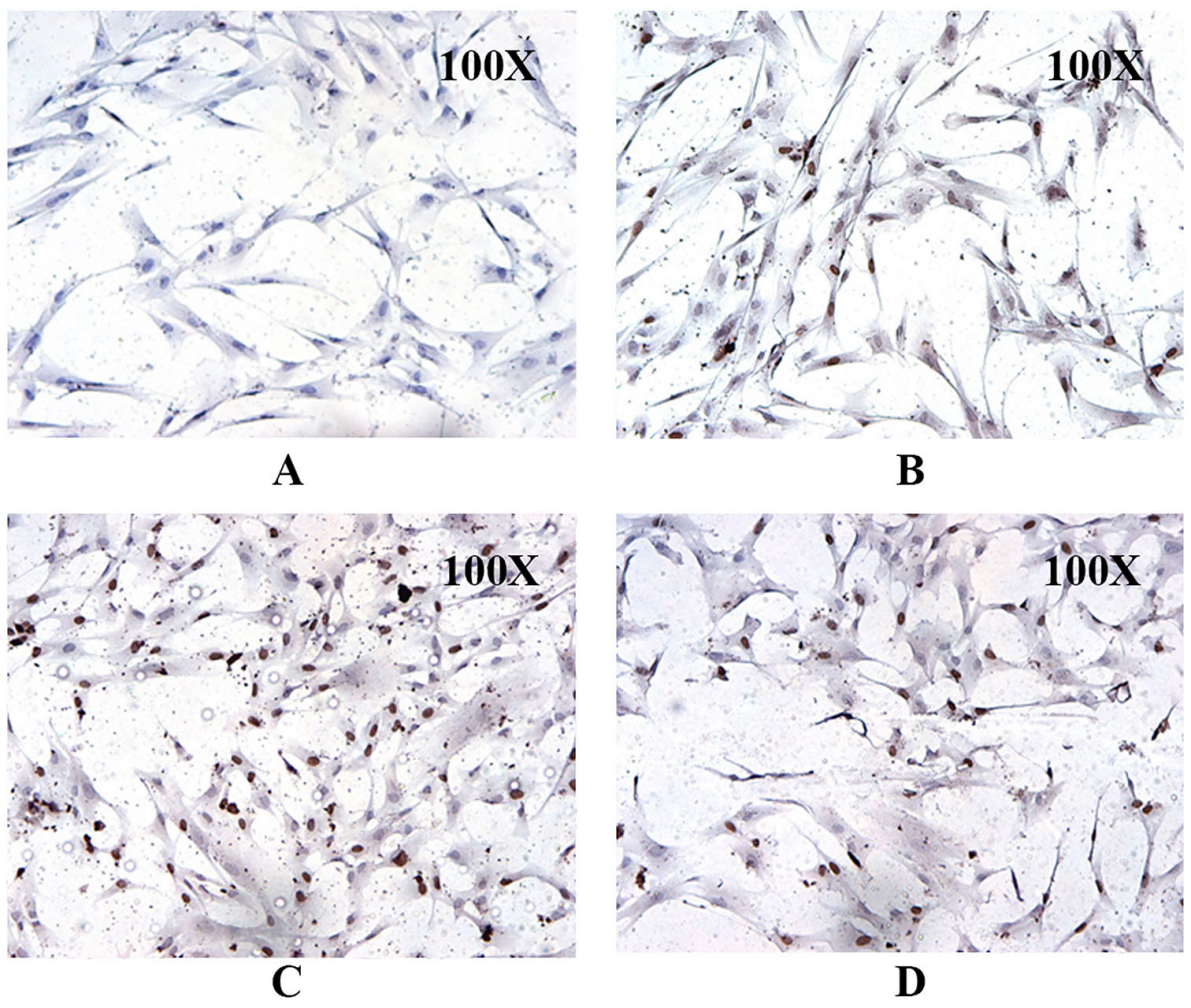
Cell cycle analysis of hUCMSC using BrdU incorporation assay at different time intervals. **A** Negative control, **B** 2 h, **C** 4 h, and **D** 6 h, respectively

The BrdU incorporation assay result displayed a higher proportion of isolated MSCs at the S phase of the cell cycle. The increase in the BrdU incorporation into the nucleus (nucleus appeared dark brown) is directly proportional to the increasing incubation time interval of MSCs with BrdU, as shown in Fig. 4. This indicates that the proportion of MSCs entering the S phase was higher with respective to the time intervals of BrDU incorporation. It confirms that the isolated UCMSCs had a higher proliferation rate with increasing time intervals (2, 4, and 6 h).

### Cytokine/chemokine expression profiling of hUCMSC CM

**Fig. 5:**
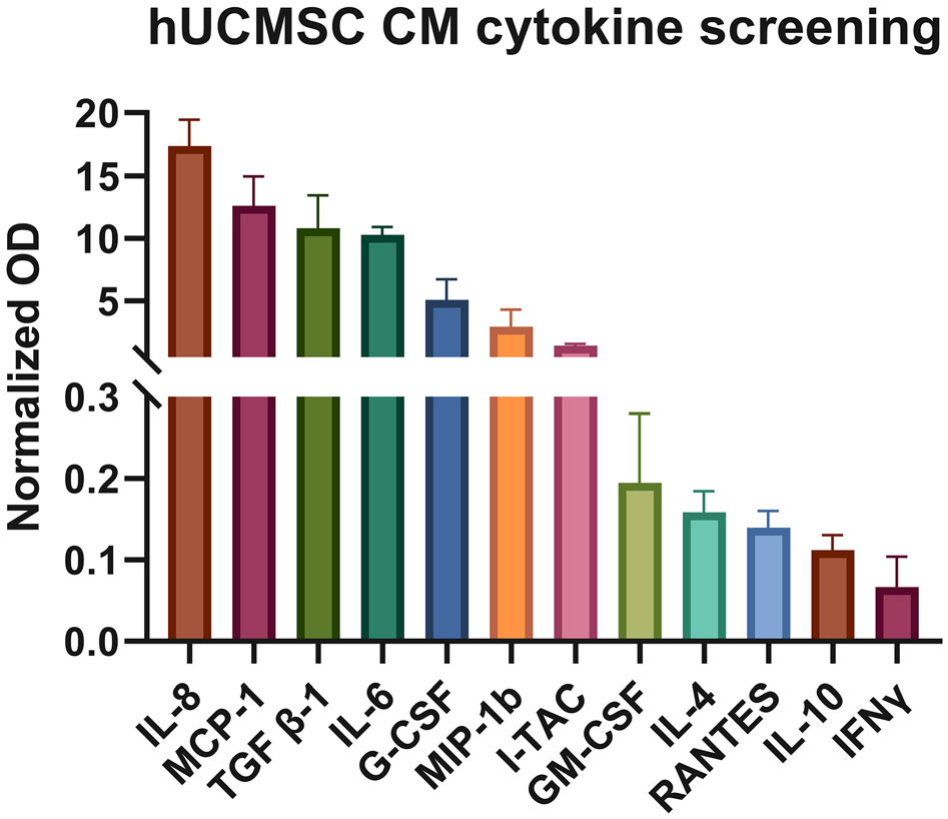
Cytokine profiling of conditioned media collected from hUCMSC at passage 1

In hUCMSC conditioned media, the expression levels of IL-8, MCP-1 (CCL2), TGF-1, IL-6, G-CSF, MIP-1 (CCL4), and I-TAC (CXCL-11) were shown to be significantly higher. The IL-8 is a crucial mediator for the mobility/migration of MSCs. Similarly, MCP-1, which promotes angiogenesis, alters cell migration, and inhibits apoptosis, was also found to be present in high quantity in the CM**.** On the other hand, GM-CSF, IL-4, RANTES (CCL5), IL-10, and IFN were observed to be less expressed in the hUCMSC conditioned media, as revealed from the ELISA results (Fig. 5).

### Proteome analysis of hUCMSC CM and functional enrichment analysis

**Fig. 6:**
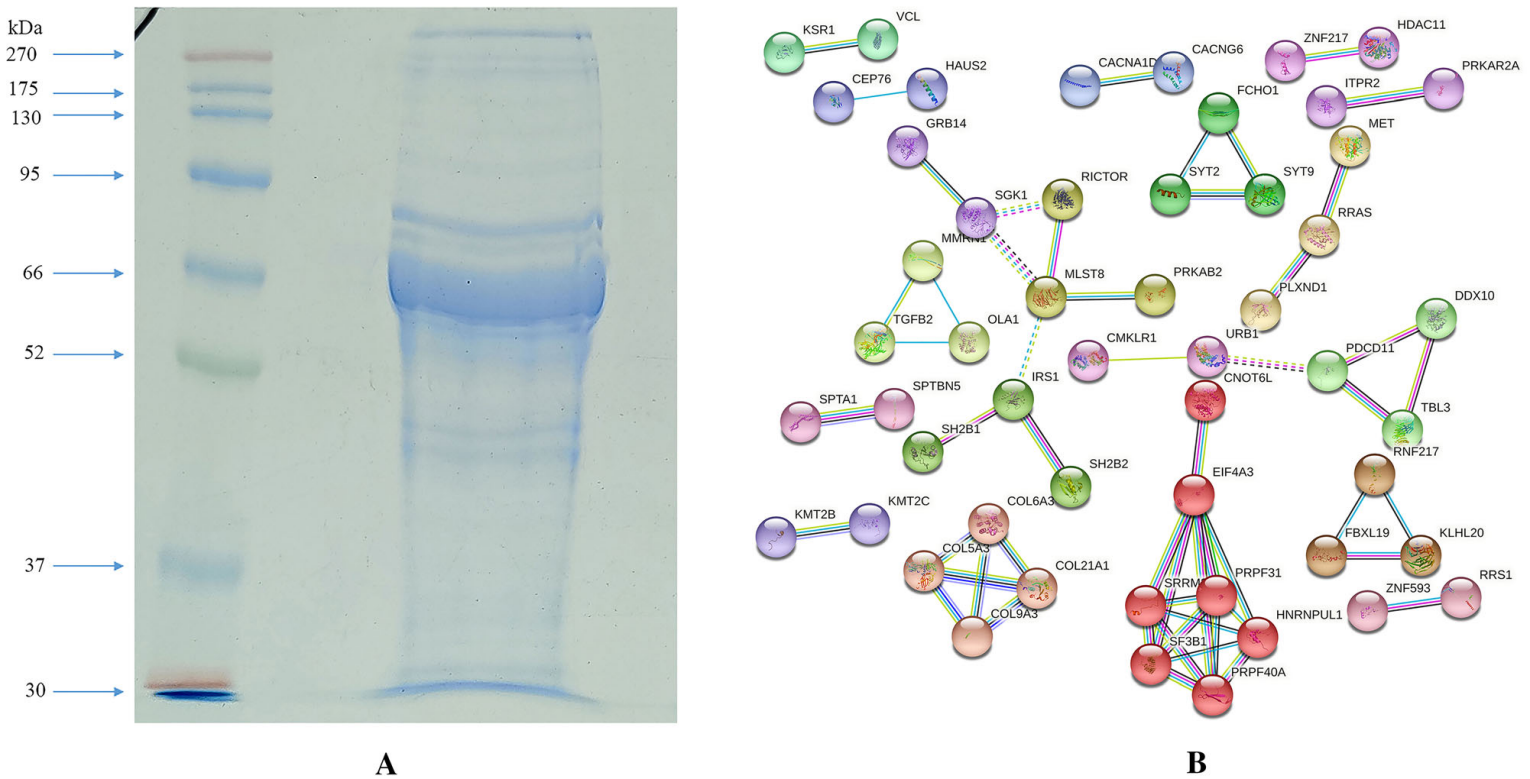
**A** hUCMSC CM protein separation by SDS PAGE, **B** Protein–protein interaction network of hUCMSC profiled proteome using STRING database

**Fig. 7:**
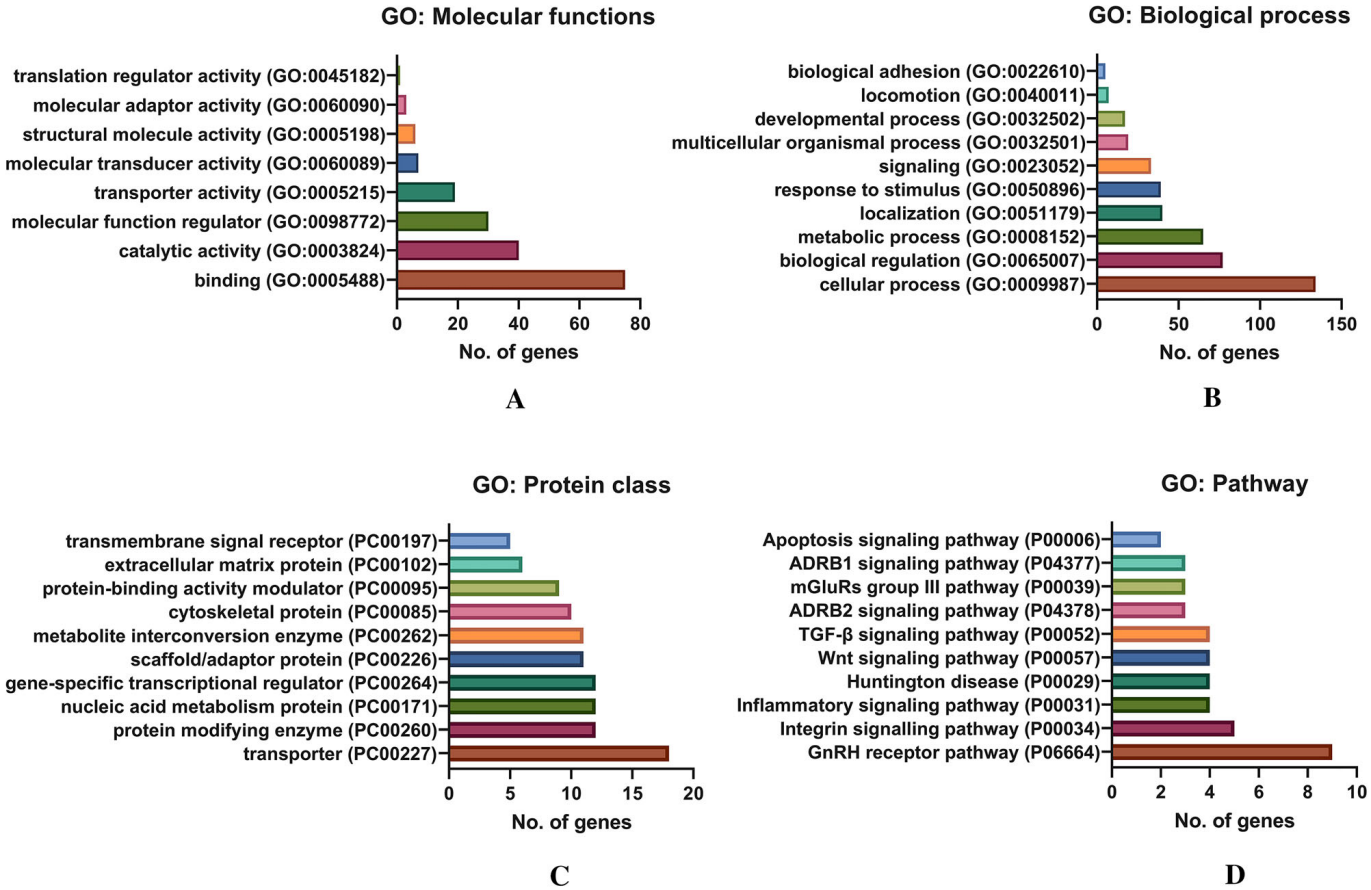
Functional enrichment analysis of identified hUCMSC CM proteins by gene ontology (GO) using PANTHER database. **A** GO analysis: Molecular functions, **B** GO analysis: Biological process, **C** GO analysis: Protein class, **D** GO analysis: Pathway analysis

The proteins were separated based on their molecular weight, as shown in Fig. 6A. The spectrum data and peptide mass were obtained from the LCMS/MS. The MASCOT analysis of these peptide spectra identified a total of 214 proteins (Supplementary Table S1). The protein–protein network association of the identified proteins was generated using the STRING database, presented in Fig. 6B. The GO molecular function analysis revealed that 41.4% of proteins identified were involved in the activity of binding (GO:0005488), 22.1% of the proteins in catalytic activity (GO:0003824), 16.6% of proteins in molecular function regulator (GO:0098772), and 10.5% in transporter activity (GO:0005215), respectively, as shown in Fig. 7A. Although observed in low percentages, the analysis also identified some proteins involved in translation regulatory activities (GO:0,045,182). The GO biological process analysis showed that 30.2% of identified proteins were involved in the cellular process (GO:0009987), 17.3% in biological regulation (GO:0065007), and 14.6% in the metabolic process (GO:0008152). As shown in Fig. 7B, the functions of some identified proteins include biological adhesion and locomotion. These biological processes are crucial for cell migration and invasion, which helps malignant cells to metastasize rapidly. Similarly, the protein class analysis classified 14.6% of the identified protein as a transporter (PC00227), 9.8% as a protein modifying enzyme (PC00260), and 9.8% as nucleic acid metabolism protein (PC00171), respectively, as displayed in Fig. 7C. Interestingly, the GO pathway analysis identified that 9.4% of proteins are actively involved in the Gonadotropin-releasing hormone receptor pathway (P06664), 5.2% in Integrin signaling pathway (P00034), 4.2% in inflammation mediated by chemokine and cytokine signaling pathway (P00031), 4.2% in *Wnt* signaling pathway (P00057), 4.2% in TGF-beta signaling pathway (P00052), and 2.1% proteins are associated with the Apoptosis signaling pathway (P00006), (Fig. 7D).

### Characterization of hUCMSC exosomes

#### Electron microscopic analysis

**Fig. 8:**
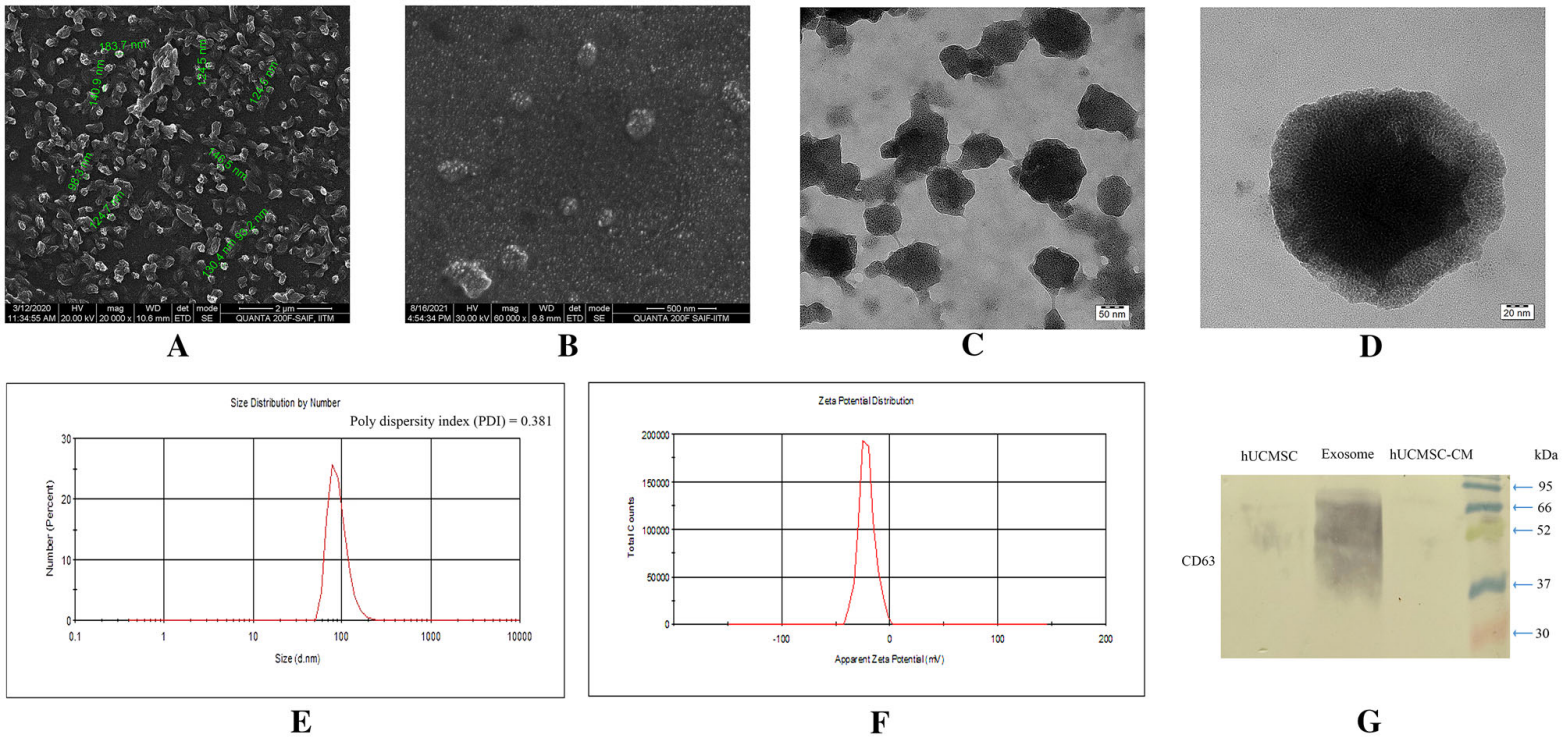
Structural analysis of hUCMSC-derived exosomes **A** (at 20,000x magnification) and **B** (at 60,000x magnification) High-resolution scanning electron microscopy (HR-SEM) image of isolated hUCMSC exosome with the measured vesicle diameter (indicated in green color). **C** (at 40,000x magnification) and **D** (at 100,000x magnification) High-resolution transmission electron microscopy (HR-TEM) image of isolated hUCMSC exosome. **E** Dynamic light scattering (DLS) spectra of exosomes for the size distribution. **F** Zeta Potential of exosomes for structural integrity analysis. **G** Western blot analysis of exosomal surface marker CD63 in hUCMSCs, hUCMSC-derived exosomes (denoted as Exosomes) and hUCMSC-CM (condition medium)

We performed structural and size distribution analysis of hUCMSC exosomes using electron microscopic imaging. HR-SEM results showed the spherical-shaped morphology of exosomes having a size distribution range from 90 to 150 nm (Fig. 8A, B). More precisely, the majority of visible exosomes appeared to have 110–140 nm in diameters, as seen in the HR-SEM results (Fig. 8A). The HR-TEM images revealed the expected morphological characteristics, including a closed intact membrane structure (Fig. 8C, D).

#### Size distribution analysis of isolated exosomes

The DLS measurements were conducted 11 times, each for 50 s. According to the zeta sizer measurements, the exosomes isolated from the hUCMSC CM were in the range of 134.8 ± 42.49 diameters in nm (d nm), consistent with previous reports [27], as shown in the spectrum peak with a PDI of 0.381, stating that the isolated exosomes are monodispersed in solution (Fig. 8E). The major scattering peak of exosomes was observed in the range of 78.82–91.28 d nm.

The structural stability of exosomes in suspension, as colloidal nanoparticles, was analyzed by studying the electrokinetic potential of exosomes. The surface charges of the exosomes are reflected by their zeta potential, and being enclosed with the lipid bilayer; the exosome surface is generally negatively charged. The observed zeta potential values (mV) were in the range of − 21.5 ± 7.25 mV, showing that the isolated exosomes were highly stable (Fig. 8F).

#### Western blot analysis

The western blot analysis revealed that the isolated extracellular vesicles highly expressed exosomal surface marker CD63 (lane denoted as Exosomes in Fig. 8G) and a very mild positive for hUCMSC lysates (lane denoted as hUCMSC in Fig. 8G) and no expression was observed on hUCMSC-CM (lane denoted as hUCMSC-CM in Fig. 8G). A smear band of CD63 expression was observed in the range of 37–66 kDa under non-reducing conditions confirming that the isolated extracellular vesicles are exosomes. The absence of the band in the hUCMSC-CM indicates that the exosomes present in the CM were in a minimal proportion that could not be detected by the western blot analysis.

### miRNA profiling of hUCMSCs and hUCMSC-derived exosomes

**Fig. 9:**
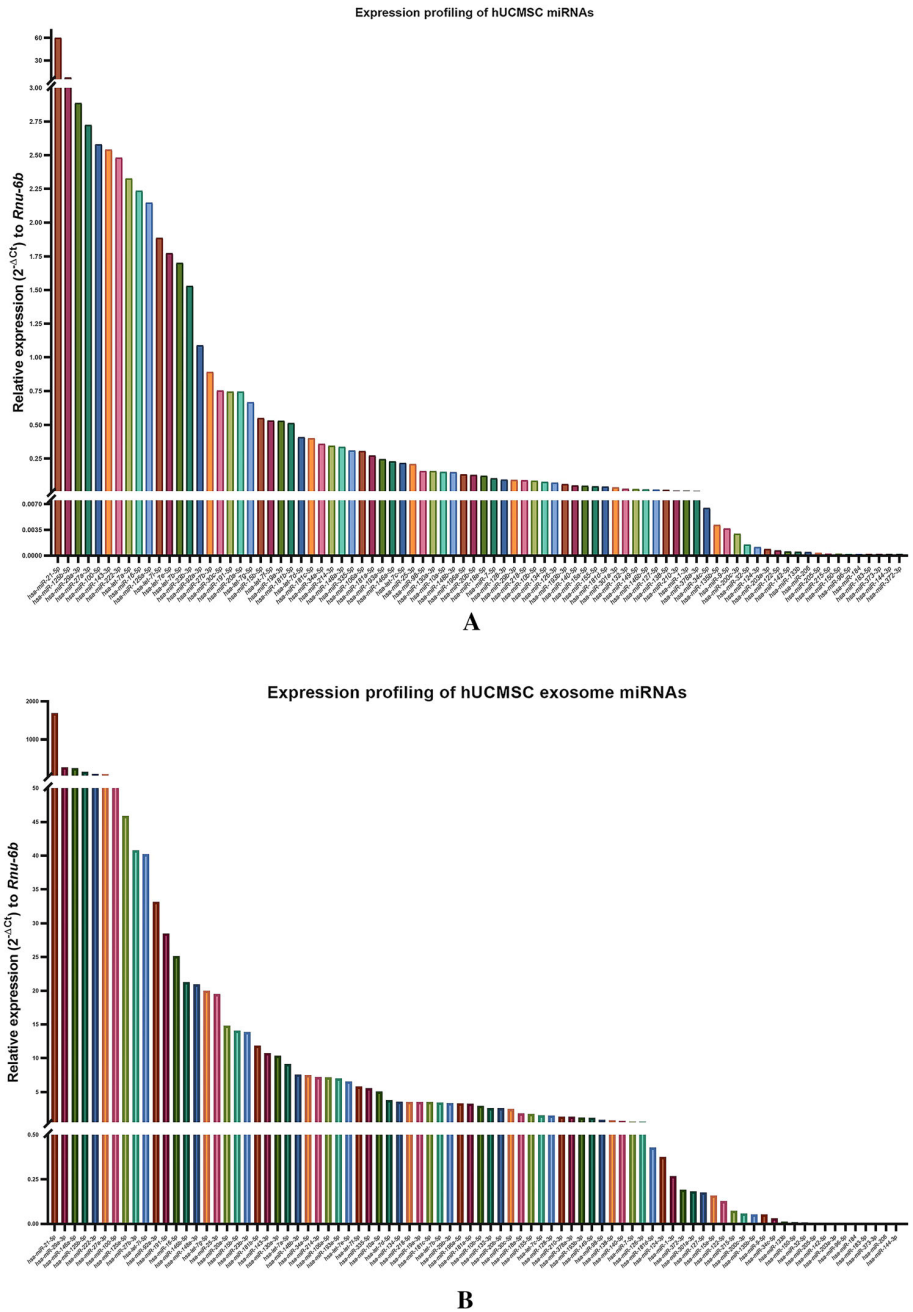
Expression analysis of miRNA using miRNA array. **A** miRNA profiling in hUCMSCs, **B** miRNA profile of hUCMSC-derived exosomes

The miRNA profiling of hUCMSC and hUCMSC-derived exosomes had provided a signature pattern of most abundantly expressed miRNAs involved in different human cancer pathways respective to the regulation of cellular proliferation, oncogenesis, apoptosis, senescence, and stemness. The result display that the expression of some miRNAs was significantly high such as *miR-21-5p, miR-125b-5p, miR-146a-5p, miR-29a-3p, miR-27a-3p, miR-100-5p, miR-143-3p, miR-222-3p* and few *let-7* family miRNAs in hUCMSCs and hUCMSC-derived exosomes. In contrast, both hUCMSCs and exosomes isolated from hUCMSCs lacked or had less expression of miRNAs like *miR-96-5p, miR-184, miR-183-5p, miR-373-3p, miR-144-3p, miR-372-3p*, and *miR-206* (Supplementary Table S2; Fig. 9A, B). Considering the expression pattern of 84 cancer-associated miRNAs, they are classified into either oncomiRs (cancer-promoting) and/or tumor suppressor miRs, yet the role of several other miRNAs is still controversial and yet to be fully elucidated. The comparative expression of upregulated and downregulated miRNAs in hUCMSCs and hUCMSC-exosomes have been represented in Fig. 9 and listed in tabular format in Supplementary Table S2.

### miRNA-mRNA interaction and functional enrichment analysis of highly expressed exosomal miRNAs

**Fig. 10:**
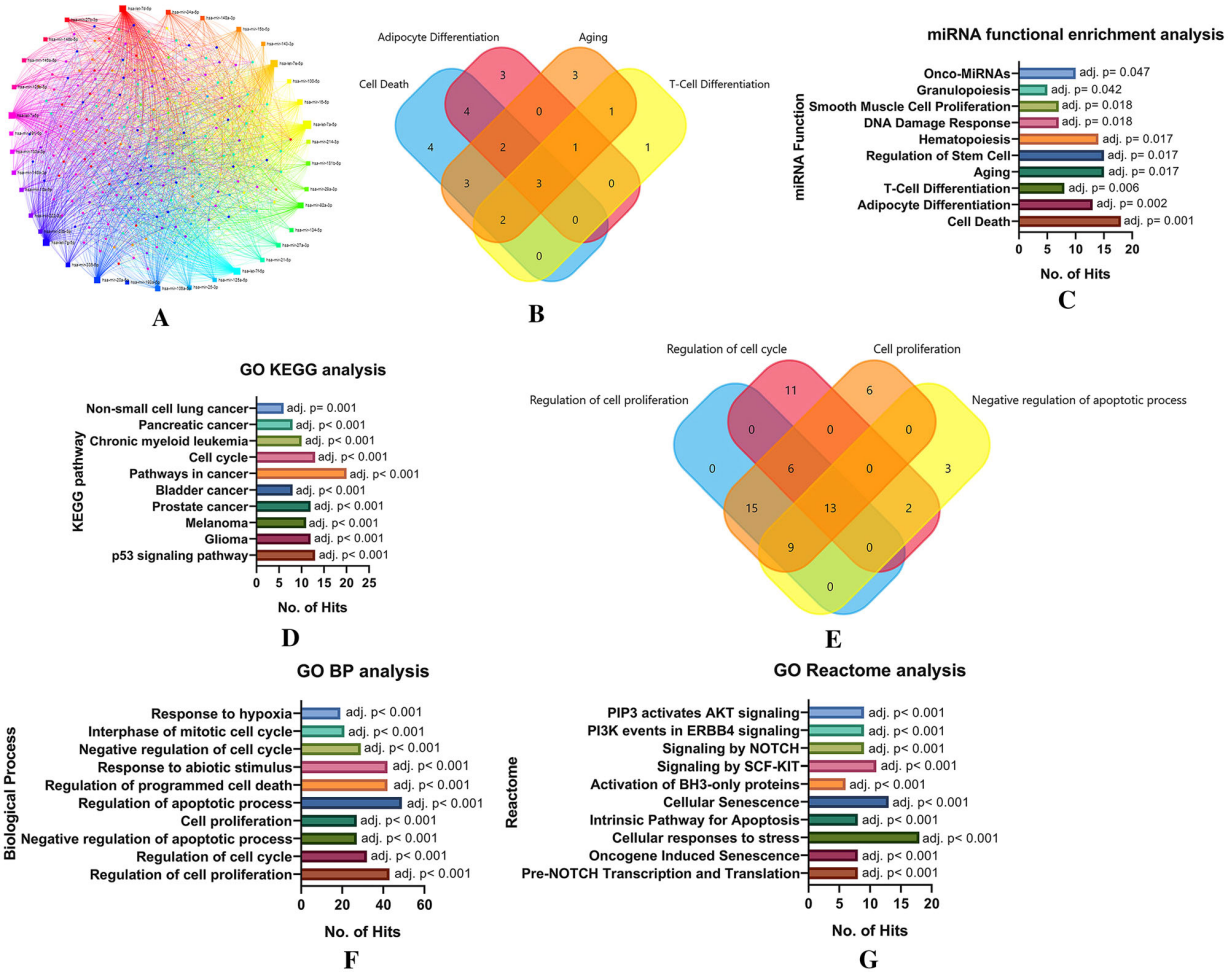
**A** miRNA –mRNA interaction network of highly expressed miRNAs in exosomes and gene ontology (GO) analysis using the miRnet database. **B** Venn diagrammatic representation of miRNAs distribution in functional enrichment analysis. **C** miRNA functional enrichment analysis of highly expressed miRNAs with top 10 function class having highly significant *p* value. **D** GO KEGG analysis of interacting nodes with highly expressed miRNAs with top 10 pathway class having highly significant *p* value. **E** Venn diagrammatic representation of interacting nodes distribution in biological process analysis. **F** GO biological process analysis of interacting nodes with highly expressed miRNAs with top 10 pathway class having highly significant *p* value; and **G** GO Reactome analysis of interacting nodes with highly expressed top 10 miRNAs having highly significant *p* values

The functional analysis has characterized the identified miRNAs based on the molecular functions, which showed that most of the miRNAs were involved in the regulation of the cell death pathway with a very high significance (adjusted *p value* of < 0.001), followed by Adipocyte and T-cell differentiation, aging, regulation of stem cell, and hematopoiesis, respectively (Fig. 10). The GO of Kyoto Encyclopedia of Genes and Genomes (KEGG) and Reactome pathway analysis displayed those genes directly interacting with the miRNAs were actively involved in cancer and/or well-known cancer-associated pathways (Fig. 10D, G). The biological process analysis of interacting genes showed a higher correlation with cell proliferation and cell cycle regulators (Fig. 10E, F), suggesting that the top highly abundant hUCMSC-derived exosomal miRNAs might have tumor suppressive characteristics and, therefore, hold promise for potential cancer therapies.

## Discussion

Mesenchymal stem cells are a distinct stromal cell population that can self-renew and exhibit multi-lineage potential, for which they have gained enormous attention in regenerative medicine [28]. They are involved in a variety of cellular activities, including tissue homeostasis maintenance and connective tissue regeneration. MSCs have numerous advantages in therapeutic applications, including their ease of availability and isolation, multilineal differentiation ability, effective immunosuppressive properties, and safety without the risk of malignancy after allogeneic cell infusion. Furthermore, isolation of MSCs from the human umbilical cord is a non-invasive and painless procedure [29]. In our study, after successful isolation, the primary human fibroblastic stromal cells were characterized based on their multi-lineage differentiation, cluster differentiation marker genes, self-renewal, and expression of stem cell marker genes for the validation as MSCs. Under appropriate culture conditions, the MSCs differentiated into adipocyte, osteocyte, and chondrocyte. The isolated fibroblasts were characterized immunophenotyping using flow cytometry and were found to be positive for the hUCMSC markers like CD44, NT5E, THY1, and ENG and negative for PTPRC and ITGB1 (CD29) as reported in our previous study (data not shown) [30]. The expression of *NT5E, ENG*, and *THY1* validates the mesenchymal stem cell-like phenotype (Fig. 3). Along with the expression of the stem cell markers such as *POU5F1, NANOG,* and *SOX9* (Fig. 3), cell cycle analysis using BrdU at different time intervals clearly showed that the isolated cells have stemness and the ability to self-renew with a higher proliferation rate [31, 32]. Because MSCs have low HLA antigens, MHC Class I, CD40, CD80, and CD86 molecules and no MHC Class II molecules, they aid in immune detection and provide an immune tolerant phenotype, allowing for the use of allogeneic cells for patients [33]. Though MSCs have a distinct self-renewal ability, they have limited replicative potential in culture, limiting them to the G1 phase of the cell cycle after several culture passages. Previous studies revealed that cell cycle events were evenly distributed in the early passages, whereas it restricts progression towards DNA synthesis and mitotic phase at the 5^th^ passage, leading to a near-complete arrest of the G1 phase at the 10th passage. Thus, restricting cell cycle progression to the S and G2/M phases activates cellular senescence (Fig. 4) [34–38]. The possibility of malignant transformation of MSCs is restricted due to their short lifespan, but this double-edged sword for therapeutic application should be further critically investigated [39].

MSCs have been demonstrated to have potent anti-inflammatory and immunomodulatory effects on nearly all innate and adaptive immune cells, exerted via several molecular pathways, including cytokine and chemokine release. When cultured, MSCs release cytokines and chemokines into the culture media, also evident from our results of cytokine profiling of the CM, and these factors may promote anti-oxidant, immunosuppressive effects, and alter immunomodulation. In the present study, high levels of IL-8, MCP-1, TGFβ-1, IL-6, GCSF, MIP-1b, and I-TAC were observed in the conditioned medium from hUCMSCs (Fig. 5). Despite the reported immunomodulatory abilities of MSCs, it has been observed that the cytokine released by MSCs promotes tumor growth and aid in epithelial-mesenchymal transition by increasing TGF-1 secretion in the TME.

MSC are drawn to tumor sites and activated by specific stimuli such as TGF-1 to produce a cancer-associated fibroblast (CAF) like phenotype [40]. Together with VEGF and angiopoietin, the paracrine activity of MSC secreted cytokines such as IL-8, TGF-1, and IL-6 promotes blood vessel development [41]. According to previous studies, MSCs actively recruit macrophages to suppress the function of T and B cells via secretion of the chemokine, monocyte chemoattractant protein 1 (MCP-1) to target the CCR2 receptor-mediated signaling, thereby promoting cancer progression [42, 43]. Earlier studies showed high levels of MSC-secreted MIP-1b (CCL4) target CCR5 in colon cancer cells, promoting invasion and metastasis [44]. It has also been reported that MSCs can inhibit angiogenesis, suppress Wnt and AKT signaling, and induce cell cycle arrest and apoptosis [45, 46]. Incoherence with cytokine profile, 4.2% of the identified proteins from proteome analysis was associated with inflammation-mediated chemokine and cytokine signaling pathway, as revealed by the GO pathway analysis (Fig. 7). The crosstalk between the MSCs and cancer cells through the cytokine, growth factors and integrins activate the oncogenic signaling pathways such as MAPK/ERK and PI3 kinase/Akt. In turn, activating growth factor receptors can increase integrin expression and avidity, resulting in improved cell adhesion [47]. Furthermore, the interaction of the Il-1β and Wnt proteins promotes cell proliferation and oncogenesis. In this regard, Il-1β activates the Wnt signaling pathway directly by inactivating GSK3β, resulting in increased β-catenin levels and promoting Wnt signaling during tumor development [48]. The earlier reports on the human mesenchymal stem cell (hMSC) secretome presented that the identified proteins were involved in the transcription and translational regulation, as transporter, as growth factor, and as transmembrane receptor [49]. The majority of human bone marrow mesenchymal stem cell (hBMMSC) secretome proteins were involved in the regulation of biological functions such as transport, transcription, cell cycle, cell adhesion, DNA damage, etc., [50]. In line with the previous reports [49–51], our findings on the hUCMSC secretome showed that the majority of proteins play an important role in the regulation of molecular functions such as binding, catalytic activity, transporter activity, and so on. Surprisingly, our findings show that the proteins secreted by hUCMSCs are deeply engaged in the GnRH receptor pathway, integrin signaling pathway, inflammation signaling pathway, Wnt, and TGF signaling cascades. In addition, the protein class analysis reveals that the major protein is a transporter, protein modifying enzyme, gene-specific transcriptional regulator, and ECM protein (Fig. 7). The correlation of proteome analysis showed that the hUCMSC is a crucial player in the TME and can inhibit or promote tumor cell growth via various types of cellular interaction [52].

Exosomes are small endocytic vesicles that are released in the extracellular space after the multivesicular bodies fuse with the plasma membrane. In our study, we have isolated and characterized exosomes obtained from the conditioned media. The size distribution of exosomes was within the range of 50–200 nm confirmed in DLS analysis, with most exosomes having a 130 nm diameter as shown in electron microscopy results (Fig. 8). Furthermore, the presence of exosomes was confirmed by the high relative expression of CD63 in isolated extracellular vesicle lysates, and very mild expression in hUCMSC lysates and whereas negligible expression was found in the CM. The overall exosome characterization result showed that isolated extracellular vesicles were found to be exosomes with a very low polydispersity index, indicating the purity of exosomes, based on DLS, Zeta potential analysis, electron microscopy, and western blot analysis. MSC secreted exosomes have been shown to easily communicate with their target cells and perform biological processes by specific receptor-ligand interactions [53, 54]. Upon endocytosis, these exosomes release messenger RNAs (mRNAs), microRNAs (miRNAs), and proteins into the accepting cells and mediate biological processes such as translation of mRNAs, miRNA-mediated silencing, and modulate other target signaling pathways [55–58].

Furthermore, several studies suggest that MSC-derived exosomes as mediators in the tumor microenvironment (TME) and play a significant role in the development of cancer, angiogenesis, and metastasis. Other studies, on the other hand, support the tumor-suppressive property of MSC-derived exosomes [59]. The molecular processes underlying MSC-derived exosomes involvement in carcinogenesis and cancer progression are not known, but a greater understanding of these mechanisms could lead to improved prognosis and tumor suppression. A recent study had demonstrated that the human bone marrow MSC-derived exosomal *miRNA-144* inhibits the progression of lung cancer and induces apoptosis [60]. Considering MSCs from various sources are thought to have distinct phenotypic characteristics, a growing number of studies have been conducted in recent years to increase our understanding of the molecular expression profile that in turn affects the properties of MSCs depending on their source of isolation. According to a comparative study on miRNA expression profiles of UCMSCs and cord blood mesenchymal stem cells (CBMSCs), miRNAs expressed in CBMSC are more actively involved in the repression of neural-related processes. Additionally, targets of highly expressed miRNAs in CBMSCs are involved in the ECM-receptor interaction, focal adhesion, and small cell lung cancer pathways [61]. Similarly, CBMSC miRNAs are involved in cellular functions such as apoptosis, cellular proliferation, cell cycle, and necrosis. Meanwhile, miRNAs from adipose tissue mesenchymal stem cells (ADMSCs) are involved in the regulation of cellular proliferation, differentiation, and migration. It has been discovered that miRNAs derived from BMMSCs regulate the proliferation of blood cells, immune cells, cell movement, leukocyte infiltration, cellular function and maintenance, apoptosis, and so on [62]. In coherence with their findings, hBMMSC derived exosomal *miR-205* inhibited prostate cancer cell proliferation, invasion, migration and induces apoptosis through the knockdown of *RHPN2* mRNA [63]. In contrast, the BMMSC exosomal *miR-19b-3p* promotes esophageal cancer cell proliferation, migration, and invasion via targeting *SOCS1* [64]. The BMMSC exosomes containing *miR-122-5p* promote proliferation and differentiation of osteoblasts by targeting the *SPRY2* and positively regulating the receptor tyrosine kinase (RTK) [65]. In a previous study, it was discovered that ADMSC and BMMSC have a similar miRNA expression pattern. However, miRNA expression in their exosomes differed significantly depending on their source as well as their cellular domain. *miR-486-5p, miR-10a-5p, miR-10b-5p, miR-191-5p, and miR-222-3p* expression was found to be significantly upregulated in ADMSC derived exosomes, whereas *miR-143-3p, miR-10b-5p, miR-486-5p, miR-22-3p, and miR-21-5p* expression was found to be overexpressed in BMMSC derived exosomes [66].

In our study, the miRNA profiling of the hUCMSC and hUCMSC-derived exosomes showed some interesting findings to our knowledge. Few miRNAs were abundantly found in exosomes compared to their cellular counterparts like *miR-146a-5p, miR-146b-5p, miR-372-5p, miR-124-3p, and miR-215-5p*. Investigating the role of these abundant exosomal miRNAs in terms of tumor promotion or suppression might provide useful knowledge about the molecular interplay between the mesenchymal stem cell and cancer cells in the TME, cell–cell communication, and epithelial-mesenchymal transition. Additionally, the varied expression of miRNAs indicates their involvement in a variety of activities in the target cells. Studies have reported that the *miR-146* was downregulated in various cancers, including non-small cell lung cancer (NSCLC), and its upregulation inhibits cellular proliferation by directly degrading the *EGFR* mRNA [67, 68]. Similarly, *miR-215* acts as a tumor suppressor by blocking the EGFR ligand *epiregulin,* and its transcriptional regulator *HOXB9* [69], which was upregulated in hUCMSC-derived exosomes, confirming their tumor suppressor activity. The present study found that *miR-124-3p* was highly upregulated in the hUCMSC exosomes compared to the hUCMSC cellular domain. *miR-124-3p* acts as a tumor suppressor in various cancers, including breast cancer, bladder cancer, gastric cancer, cervical cancer, hepatocarcinoma, etc. It was found to be downregulated in the breast cancer tissues and functions as a tumor suppressor in breast cancer by targeting *CBL* mRNA [70]. Incoherence to breast cancer restricts the cell proliferation, migration, and invasion of gastric cancer and induces apoptosis by targeting *ITGB3*, *Rac1,* and *Sp1* mRNAs [71, 72]. In hepatocarcinoma cells, *miR-124-3p* targets and degrades the *CRKL* mRNA through the miRNA-mediated gene silencing and suppresses the invasion and metastasis, thereby acting as a tumor suppressor miRNA [73].

Interestingly, several 100-fold higher level of *miR-21-5p* was found in the exosomes compared with hUCMSCs. Research suggests that *miR-21-5p* is actively involved in tumor progression, invasion, and metastasis [74, 75]. The *miR-134-5p* level was observed to be significantly high in hUCMSC-derived exosomes and hUCMSC, and a few studies have stated its involvement in tumor suppressor activity by blocking oncogenic pathways [76]. The molecular target of these miRNAs differs from one cell type to the other. For instance, the miRNAs like *miR-29a, 146a, 146b, 125b, 222* are involved in either cancer progression or tumor suppression in different cancers [77–81]. The reason behind the production and release of these miRNAs at such a higher level by hUCMSC needs to be investigated further. Using bioinformatics, we tried to analyze the roles of differentially expressed miRNAs in different cancers, and our result may provide a molecular target with higher therapeutic implications.

Furthermore, the functional enrichment of the miRNAs revealed that 18 hUCSMC-derived exosomal miRNAs play active roles in regulating cell death pathways. The analysis also showed that *miR-134-5p, miR-146a-5p, miR-23b-5p, miR-181b-5p, miR-25-3p, miR-143-3p, miR-222-3p, miR-34a-5p, miR-21-5p, and miR-16-5p* were the most abundant miRNAs that involved in the regulation of cell death. Following this, 8 miRNAs were indicated to be involved in T-cell differentiation, and 7 miRNAs in the DNA damage response (Fig. 10). The interaction nodes of these highly expressed miRNAs with the mRNA revealed that the targets of the majority of the miRNAs are genes involved in cancer-associated signaling pathways, as shown by the GO KEGG and Reactome analysis (Fig. 10). In correlation, the GO biological process analysis shows that the most abundant miRNAs target genes that act as the prime hub in regulating the cell cycle, proliferation, and cell death.

The role of MSCs in the cancer cell and tumorigenesis research has grown significantly in recent years due to their multifactorial properties of regulating EMT, tumorigenesis, apoptotic activities, and inflammatory responses. Several pioneering pieces of research paved interest in further elucidating the roles of MSC-secreted molecular factors that are indicated to be involved in the regulation of these aforementioned processes. From the present study, it is understood that the hUCMSC secretome plays a crucial role in the regulation of tumor progression. MSC-derived exosomes also exhibited the versatility and capacity to interact with numerous cell types within the immediate locality and/or distant areas to initiate specific cellular responses. Finally, the expression profile of hUCMSC and their exosomal miRNA revealed a tumor-suppressive property, suggesting that it could act as a novel therapeutic target in cancer research. Further detailed studies are required to gain a mechanistic purview of the clinical translation of exosome-based regenerative therapies. Though much research had been done for delineating the biogenesis of mesenchymal stem cell-derived exosomes but is hopeful to investigate profoundly into the potential of MSC-derived exosomes in the tumor microenvironment and provide effective treatments with the highest safety. The most challenging part for exosomes to be translated from bench side to bedside is their purification, stability, and storage. The results of this study are crucial as; subsequently we have isolated, purified, and characterized the secretome (exosome) from human primary MSCs and analyzed in detail exosome proteins and epigenetic modulators present in them, which is unique. The results elaborated here might open up novel avenues to accelerate MSC-derived exosomal therapeutic application in the clinics.

## Supplementary Information

Below is the link to the electronic supplementary material.

Supplementary Table 1: List of identified proteins from hUCMSC-derived conditioned medium using MASCOT (DOCX 33 kb)

Supplementary Table 2: miRNA expression profile of hUCMSC and hUCMSC-derived exosomes (DOCX 13 kb)
