# Supplementary material for: A Comprehensive Cancer-Associated MicroRNA Expression Profiling and Proteomic Analysis of Human Umbilical Cord Mesenchymal Stem Cell-Derived Exosomes
Source: Tissue Eng Regen Med. 2022 May 5;19(5):1013–31. doi: 10.1007/s13770-022-00450-8 (PMC9478013; doi:10.1007/s13770-022-00450-8)
Supplement: Supplementary file 1 — Supplementary Table 1: List of identified proteins from hUCMSC-derived conditioned medium using MASCOT (DOCX 33 kb) [file 13770_2022_450_MOESM1_ESM.docx]

| **Protein name** | **Description** | **Molecular Mass** |
| --- | --- | --- |
| M2OM_HUMAN | Mitochondrial 2-oxoglutarate/malate carrier protein | 34211 |
| AAKB2_HUMAN | 5'-AMP-activated protein kinase subunit beta-2 | 30397 |
| PLEC_HUMAN | Plectin | 533462 |
| ICAM5_HUMAN | Intercellular adhesion molecule 5 | 98766 |
| SKOR1_HUMAN | SKI family transcriptional corepressor 1 | 100909 |
| MAGI2_HUMAN | Membrane-associated guanylate kinase, WW and PDZ domain-containing protein 2 | 159454 |
| ATD3B_HUMAN | ATPase family AAA domain-containing protein 3B | 73098 |
| PDE4A_HUMAN | cAMP-specific 3',5'-cyclic phosphodiesterase 4A | 98709 |
| RRAS_HUMAN | Ras-related protein R-Ras | 23637 |
| PR40A_HUMAN | Pre-mRNA-processing factor 40 homolog A | 109022 |
| P63_HUMAN | Tumor protein 63 | 77649 |
| SMAG1_HUMAN | Protein Smaug homolog 1 | 80049 |
| TIM23_HUMAN | Mitochondrial import inner membrane translocase subunit Tim23 | 22100 |
| AN13B_HUMAN | Ankyrin repeat domain-containing protein 13B | 70618 |
| RB27B_HUMAN | Ras-related protein Rab-27B | 24820 |
| NAV3_HUMAN | Neuron navigator 3 | 256973 |
| CNGB3_HUMAN | Cyclic nucleotide-gated cation channel beta-3 | 92679 |
| AHNK2_HUMAN | Protein AHNAK2 | 617383 |
| NJMU_HUMAN | Protein Njmu-R1 | 45277 |
| RRP5_HUMAN | Protein RRP5 homolog | 209939 |
| CP071_HUMAN | Uncharacterized protein C16orf71 | 56160 |
| CCG6_HUMAN | Voltage-dependent calcium channel gamma-6 subunit | 28681 |
| JUND_HUMAN | Transcription factor jun-D | 35266 |
| TPC12_HUMAN | Trafficking protein particle complex subunit 12 | 79781 |
| FA53A_HUMAN | Protein FAM53A | 43186 |
| MMRN1_HUMAN | Multimerin-1 | 139221 |
| SMRD2_HUMAN | SWI/SNF-related matrix-associated actin-dependent regulator of chromatin subfamily D member 2 | 59112 |
| D39U1_HUMAN | Epimerase family protein SDR39U1 | 34840 |
| RN139_HUMAN | E3 ubiquitin-protein ligase RNF139 | 76856 |
| TBL3_HUMAN | Transducin beta-like protein 3 | 90347 |
| KMT2C_HUMAN | Histone-lysine N-methyltransferase 2C | 548270 |
| ZNF28_HUMAN | Zinc finger protein 28 | 86227 |
| DEN2A_HUMAN | DENN domain-containing protein 2A | 114694 |
| OTUD4_HUMAN | OTU domain-containing protein 4 | 124823 |
| SYT9_HUMAN | Synaptotagmin-9 | 56894 |
| ITPR2_HUMAN | Inositol 1,4,5-trisphosphate receptor type 2 | 311060 |
| CING_HUMAN | Cingulin | 136532 |
| NYAP1_HUMAN | Neuronal tyrosine-phosphorylated phosphoinositide-3-kinase adapter 1 | 88842 |
| PDE4B_HUMAN | cAMP-specific 3',5'-cyclic phosphodiesterase 4B | 83918 |
| CNGA4_HUMAN | Cyclic nucleotide-gated cation channel alpha-4 | 66299 |
| MRP6_HUMAN | Multidrug resistance-associated protein 6 | 166398 |
| CNPY3_HUMAN | Protein canopy homolog 3 | 31128 |
| TRI45_HUMAN | Tripartite motif-containing protein 45 | 66256 |
| S22A8_HUMAN | Solute carrier family 22 member 8 | 60388 |
| S36A1_HUMAN | Proton-coupled amino acid transporter 1 | 53782 |
| KI26A_HUMAN | Kinesin-like protein KIF26A | 197091 |
| MRCKB_HUMAN | Serine/threonine-protein kinase MRCK beta | 196189 |
| LIX1_HUMAN | Protein limb expression 1 homolog | 32213 |
| ZHX2_HUMAN | Zinc fingers and homeoboxes protein 2 | 92934 |
| TEX40_HUMAN | Testis-expressed sequence 40 protein | 22881 |
| PKH4B_HUMAN | Pleckstrin homology domain-containing family G member 4B | 141576 |
| TEAD4_HUMAN | Transcriptional enhancer factor TEF-3 | 48583 |
| ZMAT3_HUMAN | Zinc finger matrin-type protein 3 | 32609 |
| LATS1_HUMAN | Serine/threonine-protein kinase LATS1 | 127303 |
| SC6A7_HUMAN | Sodium-dependent proline transporter | 71662 |
| ZN282_HUMAN | Zinc finger protein 282 | 75332 |
| OBSCN_HUMAN | Obscurin | 879630 |
| SH2B2_HUMAN | SH2B adapter protein 2 | 68380 |
| F124B_HUMAN | Protein FAM124B | 51613 |
| SIA8E_HUMAN | Alpha-2,8-sialyltransferase | 44551 |
| ZFY16_HUMAN | Zinc finger FYVE domain-containing protein 16 | 171192 |
| BSN_HUMAN | Protein bassoon | 418324 |
| RAI1_HUMAN | Retinoic acid-induced protein 1 | 206191 |
| OLA1_HUMAN | Obg-like ATPase 1 | 44943 |
| IL16_HUMAN | Pro-interleukin-16 | 142976 |
| SH2B1_HUMAN | SH2B adapter protein 1 | 79774 |
| ABCCB_HUMAN | ATP-binding cassette sub-family C member 11 | 155855 |
| BAZ1A_HUMAN | Bromodomain adjacent to zinc finger domain protein 1A | 180246 |
| CSPG2_HUMAN | Versican core protein | 374585 |
| ANO2_HUMAN | Anoctamin-2 | 114695 |
| CO9A3_HUMAN | Collagen alpha-3(IX) chain | 63919 |
| GSDMB_HUMAN | Gasdermin-B | 46985 |
| DYR1A_HUMAN | Dual specificity tyrosine-phosphorylation-regulated kinase 1A | 86043 |
| AF17_HUMAN | Protein AF-17 | 113488 |
| POK3_HUMAN | HERV-K_19q12 provirus ancestral Pol protein | 109121 |
| FXL19_HUMAN | F-box/LRR-repeat protein 19 | 77426 |
| RRP44_HUMAN | Exosome complex exonuclease RRP44 | 110017 |
| IQEC2_HUMAN | IQ motif and SEC7 domain-containing protein 2 | 162549 |
| HDA11_HUMAN | Histone deacetylase 11 | 39273 |
| MCPH1_HUMAN | Microcephalin | 94301 |
| TOX2_HUMAN | TOX high mobility group box family member 2 | 51971 |
| CAC1D_HUMAN | Voltage-dependent L-type calcium channel subunit alpha-1D | 247550 |
| DYH8_HUMAN | Dynein heavy chain 8, axonemal | 517984 |
| SYNP2_HUMAN | Synaptopodin-2 | 118069 |
| MPP5_HUMAN | MAGUK p55 subfamily member 5 | 77531 |
| SPTN5_HUMAN | Spectrin beta chain, non-erythrocytic 5 | 419259 |
| PGM2L_HUMAN | Glucose 1,6-bisphosphate synthase | 71366 |
| FXYD6_HUMAN | FXYD domain-containing ion transport regulator 6 | 10706 |
| CAC1B_HUMAN | Voltage-dependent N-type calcium channel subunit alpha-1B | 264553 |
| PLD2_HUMAN | Phospholipase D2 | 106719 |
| TAC2N_HUMAN | Tandem C2 domains nuclear protein | 55763 |
| PRAX_HUMAN | Periaxin | 155149 |
| CO5A3_HUMAN | Collagen alpha-3(V) chain | 172700 |
| FOXP4_HUMAN | Forkhead box protein P4 | 73842 |
| SYNEM_HUMAN | Synemin | 173005 |
| SPTA1_HUMAN | Spectrin alpha chain, erythrocytic 1 | 281039 |
| CML1_HUMAN | Chemokine-like receptor 1 | 42921 |
| GRIK1_HUMAN | Glutamate receptor ionotropic, kainate 1 | 104712 |
| DQB2_HUMAN | HLA class II histocompatibility antigen, DQ beta 2 chain | 30595 |
| HS105_HUMAN | Heat shock protein 105 kDa | 97716 |
| MYO5A_HUMAN | Unconventional myosin-Va | 216979 |
| LIRB3_HUMAN | Leukocyte immunoglobulin-like receptor subfamily B member 3 | 69856 |
| PRKN2_HUMAN | E3 ubiquitin-protein ligase parkin | 53602 |
| HMR1_HUMAN | Major histocompatibility complex class I-related gene protein | 39626 |
| LAMP3_HUMAN | Lysosome-associated membrane glycoprotein 3 | 44661 |
| SOX13_HUMAN | Transcription factor SOX-13 | 69698 |
| KCNJ2_HUMAN | Inward rectifier potassium channel 2 | 48998 |
| IRS1_HUMAN | Insulin receptor substrate 1 | 132706 |
| SYT2_HUMAN | Synaptotagmin-2 | 47355 |
| MARCO_HUMAN | Macrophage receptor MARCO | 52968 |
| QOR_HUMAN | Quinone oxidoreductase | 35356 |
| WDR92_HUMAN | WD repeat-containing protein 92 | 40171 |
| MADD_HUMAN | MAP kinase-activating death domain protein | 184500 |
| HAUS2_HUMAN | HAUS augmin-like complex subunit 2 | 27144 |
| RNFT1_HUMAN | RING finger and transmembrane domain-containing protein 1 | 50590 |
| BD1L1_HUMAN | Biorientation of chromosomes in cell division protein 1-like 1 | 332433 |
| OX26_HUMAN | Orexigenic neuropeptide QRFP | 15046 |
| TMC3_HUMAN | Transmembrane channel-like protein 3 | 126461 |
| NCOA1_HUMAN | Nuclear receptor coactivator 1 | 157970 |
| CORO7_HUMAN | Coronin-7 | 101626 |
| LRIF1_HUMAN | Ligand-dependent nuclear receptor-interacting factor 1 | 84744 |
| LPHN3_HUMAN | Latrophilin-3 | 163591 |
| CL033_HUMAN | Putative uncharacterized protein encoded by LINC00612 | 18871 |
| FONG_HUMAN | Formiminotransferase N-terminal subdomain-containing protein | 16486 |
| DYRK2_HUMAN | Dual specificity tyrosine-phosphorylation-regulated kinase 2 | 67123 |
| CB016_HUMAN | Uncharacterized protein C2orf16 | 225780 |
| IF4A3_HUMAN | Eukaryotic initiation factor 4A-III | 47126 |
| CO6A3_HUMAN | Collagen alpha-3(VI) chain | 345167 |
| MTMR2_HUMAN | Myotubularin-related protein 2 | 73905 |
| CCAR2_HUMAN | Cell cycle and apoptosis regulator protein 2 | 103465 |
| K0947_HUMAN | Uncharacterized protein KIAA0947 | 250759 |
| TMPS9_HUMAN | Transmembrane protease serine 9 | 116115 |
| RN217_HUMAN | Probable E3 ubiquitin-protein ligase RNF217 | 61044 |
| SGK1_HUMAN | Serine/threonine-protein kinase Sgk1 | 49196 |
| SP140_HUMAN | Nuclear body protein SP140 | 100270 |
| DAPK1_HUMAN | Death-associated protein kinase 1 | 161940 |
| ZN609_HUMAN | Zinc finger protein 609 | 152352 |
| MARH5_HUMAN | E3 ubiquitin-protein ligase MARCH5 | 31781 |
| PRP31_HUMAN | U4/U6 small nuclear ribonucleoprotein Prp31 | 55649 |
| YX004_HUMAN | Putative uncharacterized protein FLJ39060 | 14172 |
| DDX10_HUMAN | Probable ATP-dependent RNA helicase DDX10 | 101168 |
| FSD1L_HUMAN | FSD1-like protein | 60225 |
| EPM2A_HUMAN | Laforin | 37647 |
| HES5_HUMAN | Transcription factor HES-5 | 18329 |
| SCOT1_HUMAN | Succinyl-CoA:3-ketoacid coenzyme A transferase 1, mitochondrial | 56578 |
| ADA2A_HUMAN | Alpha-2A adrenergic receptor | 49553 |
| PLS3_HUMAN | Phospholipid scramblase 3 | 32369 |
| SRCAP_HUMAN | Helicase SRCAP | 344996 |
| COLA1_HUMAN | Collagen alpha-1(XXI) chain | 100105 |
| RRS1_HUMAN | Ribosome biogenesis regulatory protein homolog | 41225 |
| MINT_HUMAN | Msx2-interacting protein | 403030 |
| ZN593_HUMAN | Zinc finger protein 593 | 15304 |
| DOK7_HUMAN | Protein Dok-7 | 53805 |
| TRRAP_HUMAN | Transformation/transcription domain-associated protein | 441766 |
| SON_HUMAN | Protein SON | 264063 |
| KMT2B_HUMAN | Histone-lysine N-methyltransferase 2B | 297664 |
| ZN217_HUMAN | Zinc finger protein 217 | 117081 |
| ZN550_HUMAN | Zinc finger protein 550 | 49491 |
| MANF_HUMAN | Mesencephalic astrocyte-derived neurotrophic factor | 21143 |
| IKBZ_HUMAN | NF-kappa-B inhibitor zeta | 78867 |
| SRRM2_HUMAN | Serine/arginine repetitive matrix protein 2 | 300179 |
| SMRCD_HUMAN | SWI/SNF-related matrix-associated actin-dependent regulator of chromatin subfamily A containing DEAD/H box 1 | 118126 |
| FCHO1_HUMAN | FCH domain only protein 1 | 97428 |
| VP13C_HUMAN | Vacuolar protein sorting-associated protein 13C | 424462 |
| ELYS_HUMAN | Protein ELYS | 254223 |
| CEP76_HUMAN | Centrosomal protein of 76 kDa | 75677 |
| RICTR_HUMAN | Rapamycin-insensitive companion of mTOR | 194207 |
| CNO6L_HUMAN | CCR4-NOT transcription complex subunit 6-like | 63474 |
| KLH20_HUMAN | Kelch-like protein 20 | 68880 |
| RPGR1_HUMAN | X-linked retinitis pigmentosa GTPase regulator-interacting protein 1 | 147331 |
| EMAL5_HUMAN | Echinoderm microtubule-associated protein-like 5 | 222366 |
| GRB14_HUMAN | Growth factor receptor-bound protein 14 | 61634 |
| BIG3_HUMAN | Brefeldin A-inhibited guanine nucleotide-exchange protein 3 | 243862 |
| HNRL1_HUMAN | Heterogeneous nuclear ribonucleoprotein U-like protein 1 | 96250 |
| FIS1_HUMAN | Mitochondrial fission 1 protein | 16984 |
| KAP2_HUMAN | cAMP-dependent protein kinase type II-alpha regulatory subunit | 45832 |
| KDM3B_HUMAN | Lysine-specific demethylase 3B | 193172 |
| LST8_HUMAN | Target of rapamycin complex subunit LST8 | 36537 |
| DLG2_HUMAN | Disks large homolog 2 | 97948 |
| SPAST_HUMAN | Spastin | 67497 |
| SP9_HUMAN | Transcription factor Sp9 | 49569 |
| TX13B_HUMAN | Testis-expressed sequence 13B protein | 34231 |
| SF3B1_HUMAN | Splicing factor 3B subunit 1 | 146479 |
| MISP_HUMAN | Mitotic interactor and substrate of PLK1 | 75482 |
| RYR3_HUMAN | Ryanodine receptor 3 | 557790 |
| HGD_HUMAN | Homogentisate 1,2-dioxygenase | 50616 |
| ZUFSP_HUMAN | Zinc finger with UFM1-specific peptidase domain protein | 67171 |
| PCDB4_HUMAN | Protocadherin beta-4 | 87615 |
| TBCEL_HUMAN | Tubulin-specific chaperone cofactor E-like protein | 48678 |
| PE2R4_HUMAN | Prostaglandin E2 receptor EP4 subtype | 53827 |
| DSCAM_HUMAN | Down syndrome cell adhesion molecule | 223888 |
| XIRP2_HUMAN | Xin actin-binding repeat-containing protein 2 | 383888 |
| MET_HUMAN | Hepatocyte growth factor receptor | 157779 |
| ELOV1_HUMAN | Elongation of very long chain fatty acids protein 1 | 32755 |
| F222A_HUMAN | Protein FAM222A | 47389 |
| VINC_HUMAN | Vinculin | 124292 |
| TAF1L_HUMAN | Transcription initiation factor TFIID subunit 1-like | 208711 |
| UCN3_HUMAN | Urocortin-3 | 18007 |
| TNKS2_HUMAN | Tankyrase-2 | 128492 |
| GBRR2_HUMAN | Gamma-aminobutyric acid receptor subunit rho-2 | 54401 |
| PWP2A_HUMAN | PWWP domain-containing protein 2A | 82366 |
| K22O_HUMAN | Keratin, type II cytoskeletal 2 oral | 66370 |
| TGFB2_HUMAN | Transforming growth factor beta-2 | 48572 |
| KSR1_HUMAN | Kinase suppressor of Ras 1 | 103293 |
| AEBP2_HUMAN | Zinc finger protein AEBP2 | 54946 |
| CHD7_HUMAN | Chromodomain-helicase-DNA-binding protein 7 | 337542 |
| KNOP1_HUMAN | Lysine-rich nucleolar protein 1 | 51728 |
| ZMY19_HUMAN | Zinc finger MYND domain-containing protein 19 | 26872 |
| LDB3_HUMAN | LIM domain-binding protein 3 | 78226 |
| NPA1P_HUMAN | Nucleolar pre-ribosomal-associated protein 1 | 256508 |
| ZN800_HUMAN | Zinc finger protein 800 | 76443 |
| FMN1_HUMAN | Formin-1 | 158792 |
| SLIK2_HUMAN | SLIT and NTRK-like protein 2 | 96485 |
| PLXD1_HUMAN | Plexin-D1 | 215293 |

**Supplementary table 1:** List of identified proteins from hUCMSC derived conditioned medium using MASCOT
