# Supplementary material for: A Comprehensive Cancer-Associated MicroRNA Expression Profiling and Proteomic Analysis of Human Umbilical Cord Mesenchymal Stem Cell-Derived Exosomes
Source: Tissue Eng Regen Med. 2022 May 5;19(5):1013–31. doi: 10.1007/s13770-022-00450-8 (PMC9478013; doi:10.1007/s13770-022-00450-8)
Supplement: Supplementary file 2 — Supplementary Table 2: miRNA expression profile of hUCMSC and hUCMSC-derived exosomes (DOCX 13 kb) [file 13770_2022_450_MOESM2_ESM.docx]

| **hUCMSC** | | **hUCMSC exosome** | |
| --- | --- | --- | --- |
| **Highly expressed** | **Less expressed** | **Highly expressed** | **Less expressed** |
| hsa-miR-21-5p | hsa-miR-96-5p | hsa-miR-21-5p | hsa-miR-96-5p |
| hsa-miR-125b-5p | hsa-miR-184 | hsa-miR-29a-3p | hsa-miR-184 |
| hsa-miR-29a-3p | hsa-miR-183-5p | hsa-miR-146a-5p | hsa-miR-183-5p |
| hsa-miR-27a-3p | hsa-miR-373-3p | hsa-miR-125b-5p | hsa-miR-373-3p |
| hsa-miR-100-5p | hsa-miR-144-3p | hsa-miR-222-3p | hsa-miR-206 |
| hsa-miR-143-3p | hsa-miR-372-3p | hsa-miR-27a-3p | hsa-miR-144-3p |
| hsa-miR-222-3p | hsa-miR-150-5p | hsa-miR-100-5p | hsa-miR-203a-3p |
| hsa-let-7a-5p | hsa-miR-215-5p | hsa-miR-125a-5p | hsa-miR-142-5p |
| hsa-miR-16-5p | hsa-miR-205-5p | hsa-miR-27b-3p | hsa-miR-205-5p |
| hsa-miR-125a-5p | hsa-miR-206 | hsa-let-7i-5p | hsa-miR-32-5p |
| hsa-let-7i-5p | hsa-miR-133b | hsa-miR-92a-3p | hsa-miR-150-5p |
| hsa-let-7e-5p | hsa-miR-142-5p | hsa-miR-191-5p | hsa-miR-133b |
| hsa-let-7b-5p | hsa-miR-122-5p | hsa-miR-16-5p | hsa-miR-34c-5p |
| hsa-miR-23b-3p | hsa-miR-203a-3p | hsa-miR-146b-5p | hsa-miR-9-5p |
| hsa-miR-92a-3p | hsa-miR-124-3p | hsa-miR-148a-3p | hsa-miR-135b-5p |
| hsa-miR-27b-3p | hsa-miR-32-5p | hsa-let-7g-5p | hsa-miR-200c-3p |
| hsa-miR-30c-5p | hsa-miR-200c-3p | hsa-miR-25-3p | hsa-miR-215-5p |
| hsa-miR-191-5p | hsa-miR-9-5p | hsa-miR-20a-5p | hsa-miR-122-5p |
| hsa-miR-20a-5p | hsa-miR-135b-5p | hsa-miR-15b-5p | hsa-miR-15a-5p |
| hsa-let-7g-5p | hsa-miR-34c-5p | hsa-miR-23b-3p | hsa-miR-127-5p |

**Supplementary Table 2: List of most abundantly expressed and less expressed miRNA in hUCMSC and its exosomes**
